# Anion Exchange Membrane Seawater Electrolysis at 1.0 A cm^−2^ With an Anode Catalyst Stable for 9000 H

**DOI:** 10.1002/advs.202416661

**Published:** 2025-03-28

**Authors:** Jian Du, Zhiheng Li, Linqin Wang, Yunxuan Ding, Wentao Ye, Wenxing Yang, Licheng Sun

**Affiliations:** ^1^ Center of Artificial Photosynthesis for Solar Fuels and Department of Chemistry, School of Science and Research Center for Industries of the Future Westlake University 600 Dunyu Road Hangzhou Zhejiang Province 310000 China; ^2^ Division of Solar Energy Conversion and Catalysis at Westlake University Zhejiang Baima Lake Laboratory Co., Ltd. Hangzhou Zhejiang Province 310000 China

**Keywords:** anion exchange membrane, dynamic equilibrium, interlayer, OER electrocatalysts, seawater electrolysis

## Abstract

Hydrogen production through seawater electrolysis is promising but challenging due to severe anode corrosion by chlorine (Cl^−^) ions. Herein, a corrosion‐resistant NiFe layered double hydroxide electrode (CAPist‐S1) is reported as a high‐performance electrocatalyst for seawater oxidation, achieving an industrial‐level current density of 1.0 A cm^−2^ at overpotentials of 200 and 220 mV in alkaline simulated (1 M KOH + 0.5 M NaCl) and natural (1 M KOH + seawater) seawater, respectively, along with extraordinary long‐term stability over 9000 h under 1.0 A cm^−2^ in alkaline natural seawater. A dense NiFe LDH interlayer generated between the NiFe LDH nanosheets and metal substrate is found to efficiently retard the penetration of Cl^−^ ions to the substrate surface, improving the resistance to Cl^−^ ions corrosion. Furthermore, this dense interlayer is an essential prerequisite for establishing a dynamic equilibrium between Fe leaching and redeposition over the in situ formed FeOOH, and this dynamic equilibrium can in turn stabilize the dense interlayer, maintaining the activity of CAPist‐S1 during prolonged electrolysis. Using CAPist‐S1 in an anion exchange membrane (AEM) seawater electrolyzer, the obtained electrolyzer stably functions over 700 h at 1.0 A cm^−2^ under room temperature, indicating promising prospects for industrial seawater electrolysis application.

## Graphical abstract

The CAPist‐S1 catalyst containing dense interlayer structure is synthesized by one‐step autologous method. The dense NiFe LDH interlayer inhibits the Cl^−^ ions penetration onto metal substrate, significantly improving the corrosion resistance. The obtained catalyst requires an overpotential of 220 mV at 1.0 A cm^−2^ and exhibits excellent durability over 9000 h at 1.0 A cm^−2^ in alkaline natural seawater.

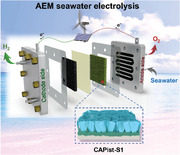

## 1 Introduction

Green hydrogen (H_2_) originating from water electrolysis can serve as a valuable energy carrier to address the energy crisis and environment pollution.^[^ ^1^, ^2^ ^]^ The current technologies for water electrolysis such as alkaline water electrolysis (AE), proton exchange membrane (PEM) water electrolysis, and anion exchange membrane (AEM) water electrolysis usually require a large amount of freshwater as feedstock, which conflicts with freshwater resources needed by human life.^[^ ^3^, ^4^ ^]^ Considering the abundance of seawater, which accounts for 96.5% of the total water on earth,^[^ ^5^ ^]^ the use of seawater as an alternative to conventional freshwater for industrial‐grade water electrolysis is promising for alleviating the freshwater shortage. Currently, the indirect seawater electrolysis by coupling the mature desalination system with the conventional electrolyzer has been proposed to be a promising solution for large‐scale hydrogen production in ocean due to the much lower cost of desalination in comparison with electrolysis.^[^ ^6^ ^]^ Nevertheless, the cost gap between electrolysis and desalination will be largely narrowed with the rapid development of electrocatalysts, electrolyzer design and electrolysis technology, as well as the low electricity cost from sustainable energy such as solar and offshore wind energy.^[^ ^7^ ^]^ Moreover, in alkaline electrolysis system including alkaline electrolyzer and AEM electrolyzer, the overall cost proportion of desalination system will exceed 7%,^[^ ^3^, ^8^ ^]^ imposing considerable costs. Therefore, the development of direct seawater electrolysis is necessary.

However, the plenty of chloride (Cl^−^) ions in seawater is a fundamental barrier to direct seawater electrolysis due to the electrochemical competition between the oxygen evolution reaction (OER) and the chlorine evolution reaction (ClER) at the anode side, which can lower the OER selectivity and result in the formation of corrosive Cl^−^‐related compounds.^[^ ^9^, ^10^, ^11^ ^]^ According to the Pourbaix diagram proposed by Peter Strasser and co‐workers,^[^ ^12^ ^]^ the maximum equilibrium potential difference of ClER to OER in alkaline conditions is ≈480 mV. In this regard, seawater electrolysis is generally carried out in the alkaline medium to possibly exclude the interference of ClER, thus achieving 100% OER selectivity. Consequently, developing high‐performance OER electrocatalysts is urgently needed for highly efficient H_2_ production through alkaline seawater electrolysis.

**Figure 1:**
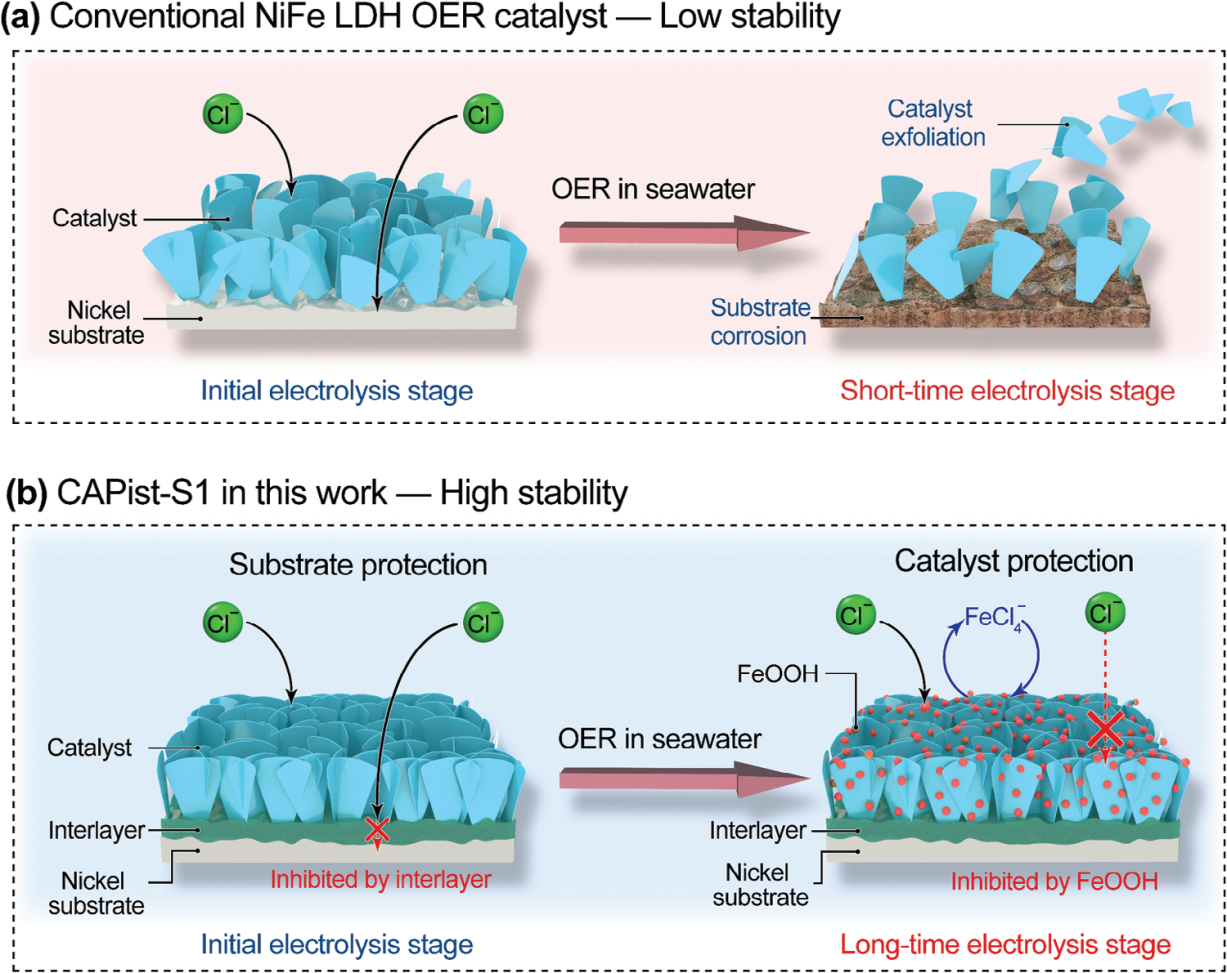
a) Illustration of Cl^−^ corrosion of conventional NiFe LDH. b) Schematic diagram of the corrosion resistance principle for the CAPist‐S1 designed in this work.

First‐row 3d transition metal composites such as NiFe‐based materials have emerged as favorable OER electrocatalysts in alkaline seawater electrolysis owing to their earth abundance, cost‐efficiency, excellent catalytic activity, and superior selectivity.^[^ ^13^, ^14^, ^15^ ^]^ Nevertheless, the lifetime of these NiFe‐based catalysts hardly satisfies the commercial demands at industrial‐level current density (≥ 1.0 A cm^−2^) because of the unavoidable anode corrosion caused by Cl^−^ ions (**Figure** **1**a), thereby restraining their practical application.^[^ ^16^, ^17^, ^18^ ^]^ To overcome this problem, the construction of anion‐rich protective layers on the surface of NiFe‐based catalysts has been employed to repulse Cl^−^ ions away from the catalyst surface through electrostatic force.^[^ ^19^, ^20^, ^21^ ^]^ However, due to the instability of these anion layers, the long‐term durability of the modified catalysts especially under industrial‐level current densities is still unsatisfactory.^[^ ^22^ ^]^ Facing this huge challenge, it is essential to exploit other strategies for enhancing the corrosion resistance of NiFe‐based electrocatalysts.

Previous studies revealed that the multilayered OER electrodes, in which the NiFe‐based alloy interlayers as cores and the in situ formed NiFe (oxy)hydroxides as shells, have shown promising catalytic performance for alkaline seawater electrolysis.^[^ ^23^, ^24^ ^]^ The surface NiFe (oxy)hydroxides as OER active species draw large current densities, while the alloy interlayers as Cl^−^ blocking materials suppress the chloride corrosion at the substrate surface, enabling the high resistance to Cl^−^ ions corrosion. Moreover, in contrast to the conventional blocking layers covering the surface of the catalyst layer, which may mask partial catalytic sites and decrease the overall OER activity,^[^ ^25^, ^26^ ^]^ the NiFe‐based alloy interlayers laying between the catalyst layers and substrates would not reduce the number of exposed surface sites, ensuring the OER activity. In response to this design concept, a nano‐micro NiOOH@Ni(OH)_2_ heterostructure is functionalized with SO_x_ group to form a cation‐selective protective layer, which can impede the Cl^−^ ions diffusion to catalyst interface and simultaneously abstract hydrogen from reaction intermediates, thus resulting in improved selectivity and corrosion resistance of the electrode.^[^ ^27^ ^]^ Similarity, by coating the sulfonated conjugated microporous polymer poly[1,3,5‐tris(4‐diphenylaminophenyl)benzene] (SPTTPAP) on IrO_2_ surface, the obtained SPTTPAP/IrO_2_ catalyst combines electron conductivity of the organic framework and proton conductivity of ‐SO_3_H group, and weakens the Cl^−^ adsorption, achieving efficient and stable seawater electrolysis.^[^ ^28^ ^]^ In addition, our group recently reported a NiFe LDH‐based OER electrocatalyst (named as CAPist‐L1), in which a dense NiFe LDH interlayer structure ensured excellent OER catalytic stability over 21 months at 1.0 A cm^−2^ in 1 M KOH.^[^ ^29^ ^]^ The presence of this dense interlayer was demonstrated to anchor the catalyst layer firmly onto the metal substrate, enabling high mechanical stability of the resultant electrocatalysts. Inspired by the above merits, the design of NiFe‐based electrocatalysts with interlayer structure may be a promising strategy to realize long‐term stability for alkaline seawater electrolysis.

In response to this thought, we report here the fabrication of compact NiFe layered double hydroxide (LDH) nanosheets with a flower‐like structure integrated on the macroporous nickel foam (NF) skeleton (CAPist‐S1) through the autologous growth. In this approach, NF serves as both a substrate and Ni source for the growth of NiFe LDH nanosheets, enabling the firm anchor of the catalyst layer on the substrate like a tree rooted in the ground. Moreover, a dense NiFe LDH interlayer generated within the inner structure of CAPist‐S1 can efficiently retard the penetration of Cl^−^ ions to the substrate surface, resulting in improved corrosion resistance to Cl^−^ ions. The dense interlayer guarantees the electrode stability in the initial OER electrolysis stage, allowing the establishment of a dynamic equilibrium between Fe leaching and redeposition over the in situ formed FeOOH. This dynamic equilibrium can in turn stabilize the structure of the dense NiFe LDH interlayer, maintaining the activity of CAPist‐S1 during prolonged OER of seawater electrolysis (Figure 1b). Specifically, the resultant CAPist‐S1 electrode exhibits ultralow overpotentials of 200 and 220 mV to afford the industrial‐level current density of 1.0 A cm^−2^ in alkaline simulated (1 M KOH + 0.5 M NaCl) and natural (1 M KOH + seawater) seawater, respectively. Furthermore, the obtained electrode shows excellent long‐term stability at 1.0 A cm^−2^ over a period of 9000 h in alkaline natural seawater. The remarkable OER performance of the CAPist‐S1 catalyst is also demonstrated in an AEM seawater electrolyzer, where a low cell voltage of 1.91 V is required to reach the current density of 1.0 A cm^−2^ when using alkaline natural seawater as the electrolyte. Impressively, the resultant AEM seawater electrolyzer with CAPist‐S1 can sustain an industrial‐level current density of 1.0 A cm^−2^ for over 700 h at room temperature, indicating its industrial prospect for large‐scale hydrogen production through seawater electrolysis. This work not only deals with the key challenge in seawater electrolysis by mitigating Cl^−^ ions corrosion to anode, significantly enhancing the catalytic stability of NiFe‐based catalysts under industrial‐level current densities, but also provides an easy and sustainable synthetic route for the large‐scale production of anode materials. Looking forward, this design concept of catalyst involving in this work may be extended to other electrochemical reactions such as hydrogen evolution reaction (HER) and CO_2_ reduction, further advancing the zero‐carbon target.

## 2 Results and Discussion

### 2.1 Preparation and Characterization of CAPist‐S1

**Figure 2:**
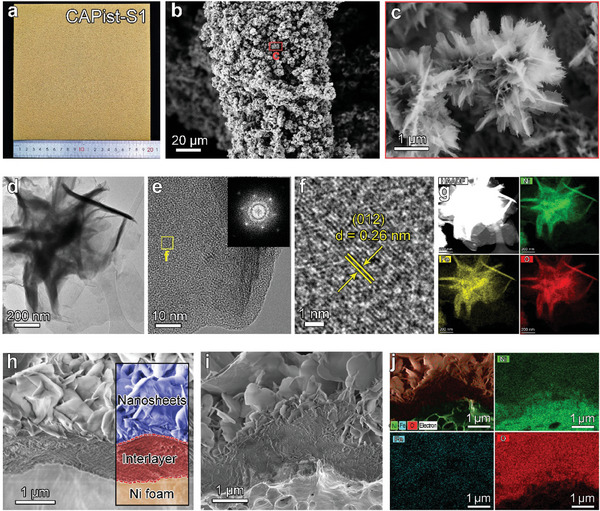
a) Digital photography of CAPist‐S1 with a size of 20 cm × 20 cm. b,c) SEM images of CAPist‐S1 under different magnifications. d–f) TEM and HRTEM images of CAPist‐S1. The inset in Figure 1e shows the SAED pattern in the red frame region. g) STEM image and corresponding elemental mappings of LDH nanosheets. h) Cross‐sectional SEM image of CAPist‐S1. (i‐j) SEM image and corresponding EDS elemental mapping analysis of the inner structure for CAPist‐S1.

The synthetic process of CAPist‐S1 is illustrated in Figure S1 (Supporting Information). Briefly, a piece of acid‐treated NF was immersed in an isopropanol and deionized water mixed solution containing Ni^2+^ and Fe^2+^ cations under the atmosphere at room temperature. After reaction for 24 h, the color of the metal NF changes from silver grey to yellow (Figure S2, Supporting Information), indicating the successful growth of the catalyst layer on the NF skeleton. In this process, Fe^2+^ was oxidized to Fe^3+^ with the aid of oxygen and water in the mixed solution, which can subsequently etch the NF substrate to release Ni^2+^ cations. The produced Fe^3+^ and Ni^2+^ ions act as metal centers and react with OH^−^ ions from oxygen reduction, forming NiFe LDH roots. With increasing reaction time, the additional Fe^3+^ and Ni^2+^ ions in the liquid phase can lead to the improved growth of nanosheets along the initial NiFe LDH roots, and finally the formation of the interlaced and uniformly compact nanosheet arrays on the NF skeleton (Figure S3, Supporting Information). This method also allows large‐scale synthesis of CAPist‐S1 electrodes with arbitrary sizes, for example, 20 cm × 20 cm (**Figure** **2**a).

The structure of CAPist‐S1 was identified based on the X‐ray diffraction (XRD) analysis (Figure S4a, Supporting Information), in which the three peaks found at 11.5°, 23.1° and 34.4° agree well with the (003), (006) and (012) planes of NiFe LDH (JCPDF NO. 49–0188) and the other three strong peaks at 44.5°, 51.9° and 76.3° are attributed to metallic NF substrate (JCPDF NO. 04–0850). The Raman spectrum of the sample in Figure S4b (Supporting Information) displays two representative bands located at 455.4 and 537.3 cm^−1^, which can be assigned to the e_g_ bending mode and A_1g_ stretching mode of Ni‐O in LDH, respectively, further confirming the successful formation of the NiFe LDH structure in CAPist‐S1.^[^ ^30^ ^]^ The morphology of the as‐prepared CAPist‐S1 was characterized by scanning electron microscopy (SEM) and transmission electron microscopy (TEM). As shown in SEM images (Figure 2b,c), the NF surface becomes rough compared with pristine NF (Figure S3a, Supporting Information), and the interlaced nanosheets with a flower‐like morphology uniformly cover the NF skeleton. The nanosheet morphology of CAPist‐S1 was also demonstrated in the TEM image (Figure 2d). Figure 2e,f shows high‐resolution transmission electron microscopy (HRTEM) images of CAPist‐S1, where the lattice spacing of 0.26 nm corresponds to the (012) plane of NiFe LDH.^[^ ^31^, ^32^ ^]^ The selected area electron diffraction (SAED) pattern in the inset of Figure 2e shows obvious diffraction rings, further indicating the crystalline nature of the as‐prepared catalyst. High‐angle annular dark field‐scanning transmission electron microscope (HAADF‐STEM) image and corresponding elemental mapping in Figure 2g suggest the homogenous distribution of Ni, Fe, and O within LDH nanosheets. Although CAPist‐S1 differs from our previously reported OER electrocatalyst (CAPist‐L1) referring to the surface morphology,^[^ ^29^ ^]^ a dense interlayer with the thickness of 1∽2 µm can be also observed in CAPist‐S1 according to the cross‐sectional SEM image (Figure 2h). Corresponding energy dispersive X‐ray (EDX) mapping analysis indicates the existence and uniform distribution of Ni, Fe, and O across the catalyst layer (Figure 2i,j), confirming the formation of a dense NiFe LDH interlayer within the CAPist‐S1 electrode. The formation mechanism and detailed characterization of the dense NiFe LDH interlayer have been clarified in our previous work.^[^ ^29^ ^]^ The loading mass of NiFe LDH catalyst on NF in CAPist‐S1 was determined to be 3.65 mg cm^−2^. Additionally, the molar ratio of Ni/Fe in CAPist‐S1 was calculated to be 3.6:1 based on the inductively coupled plasma atomic emission spectroscopy (ICP‐AES) analysis (Table S1, Supporting Information).

The composition and surface chemical valence state of CAPist‐S1 were investigated by X‐ray photoelectron spectroscopy (XPS). In Figure S5a (Supporting Information), the XPS survey spectrum shows the characteristic peaks of Ni, Fe, and O, which corresponds well with the HAADF‐STEM mapping analysis results. The high‐resolution Ni 2p XPS spectrum exhibits two spin‐orbit peaks of Ni 2p_3/2_ and Ni 2p_1/2_ at the binding energies of 856.0 and 873.4 eV, along with two satellite peaks at 862.0 and 880.1 eV (Figure S5b, Supporting Information), indicating the valence state of Ni on the CAPist‐S1 surface is predominantly +2.^[^ ^33^, ^34^ ^]^ Besides, the other two peaks at binding energies of 857.7 and 874.9 eV manifest the existence of Ni^3+^.^[^ ^30^, ^35^ ^]^ The Fe 2p XPS spectrum (Figure S5c, Supporting Information) displays two characteristic peaks at 713.1 and 726.1 eV, which can be ascribed to Fe^3+^.^[^ ^36^ ^]^ The O 1s spectrum in Figure S5d (Supporting Information) can be deconvoluted into two peaks, which arise from M‐O/M‐OH (531.4 eV) and surface‐absorbed water (532.4 eV), respectively.^[^ ^37^ ^]^ Overall, the XPS measurements indicate the formation of the NiFe LDH layers.

### 2.2 Electrochemical Properties of CAPist‐S1 for Seawater Oxidation

Since no other species were identified from the above characterization, the NiFe LDH layers in CAPist‐S1 are undoubtedly the only active species for OER. Thus, to demonstrate the advantages of this autologous growth in the catalytic performance, a conventional NiFe LDH electrode was prepared using a solvothermal method for comparison. The corresponding morphology and structural characterization of NiFe LDH electrode are presented in Figure S6 (Supporting Information). The loading mass of NiFe LDH catalyst on NF in NiFe LDH electrode was determined to be 3.38 mg cm^−2^. The OER activity of the NiFe LDH electrode in alkaline medium is superior to that of most reported NiFe LDH electrodes synthesized from various strategies such as hydrothermal, electrodeposition and co‐precipitation, etc. (Figure S7 and Table S2, Supporting Information).^[^ ^22^, ^38^, ^39^ ^]^ This result indicates that the as‐prepared NiFe LDH electrode is not a low‐performance reference sample specifically chosen to accentuate performance of the targeted CAPist‐S1.

**Figure 3:**
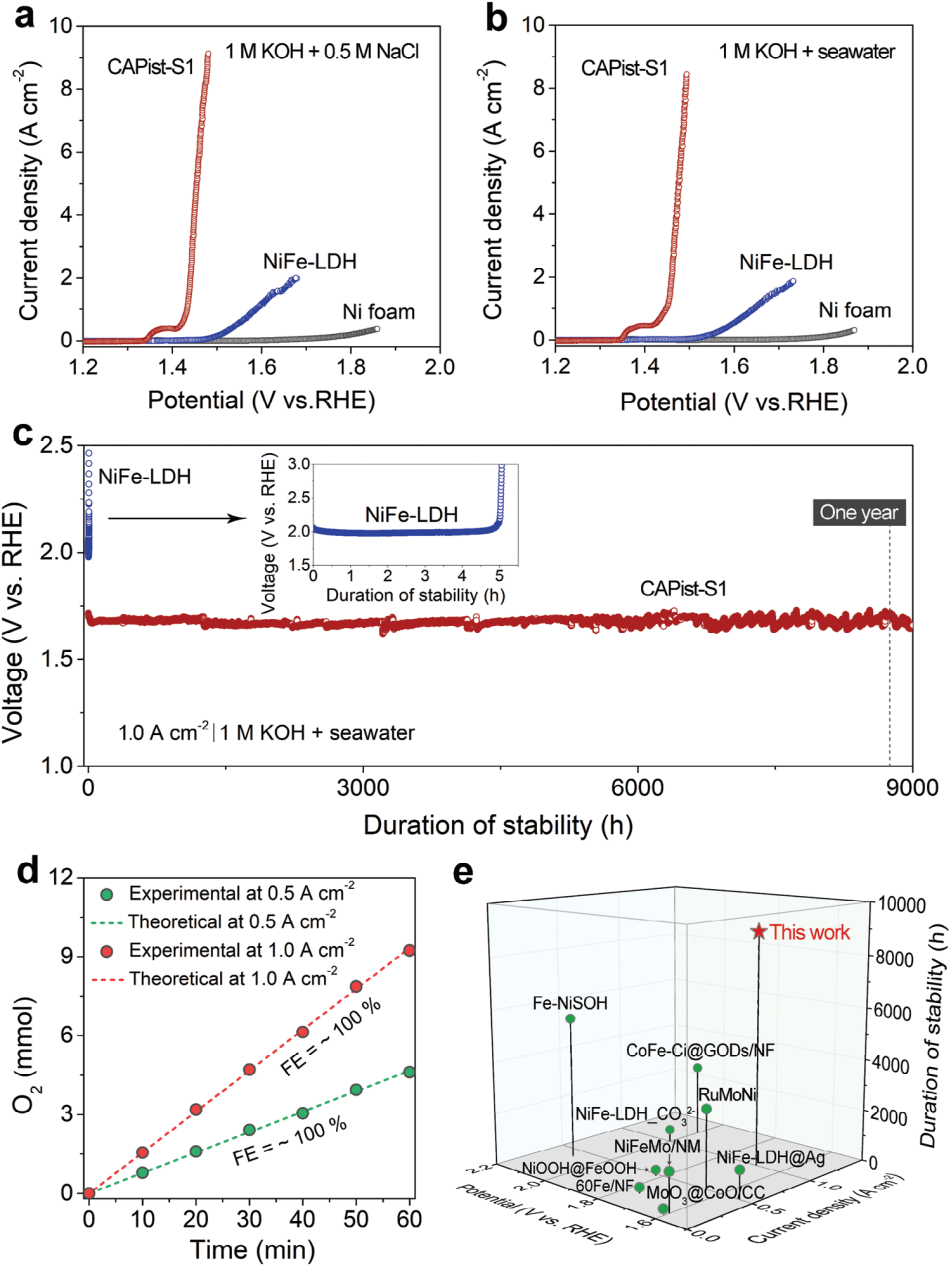
LSV curves of NF, NiFe LDH and CAPist‐S1 corrected with iR compensation in alkaline a) simulated seawater and b) natural seawater at a scan rate of 5 mV s^−1^. c) Chronopotentiometric tests of CAPist‐S1 and NiFe LDH in alkaline natural seawater at a current density of 1.0 A cm^−2^. The inset shows the enlarged stability test curve of NiFe LDH. d) Faradaic efficiency tests of CAPist‐S1 in alkaline natural seawater at the constant current densities of 0.5 and 1.0 A cm^−2^, respectively. e) Summary of recently reported electrocatalysts for alkaline simulated and natural seawater oxidation.^[^ ^4^, ^6^, ^15^, ^18^, ^21^, ^40^, ^41^, ^42^, ^43^ ^]^ Detailed information can be found in Table S3 (Supporting Information).

The electrochemical OER performance of the as‐prepared catalyst was evaluated in a typical three‐electrode setup with the use of CAPist‐S1 as the working electrode, platinum (Pt) mesh as the counter electrode and Hg/HgO as the reference electrode, respectively. Blank NF and NiFe LDH were employed as the reference samples. The OER performance of the three catalysts was first measured in alkaline simulated seawater at room temperature. According to the manual iR‐corrected linear scan voltammetry (LSV) curves shown in **Figure** **3**a, CAPist‐S1 shows the best catalytic activity among all the samples over the measured potential range. Ultralow overpotentials of 190 and 200 mV are required for CAPist‐S1 to achieve the current densities of 0.5 and 1.0 A cm^−2^, respectively, which outperform those of NiFe LDH (313 mV at 0.5 A cm^−2^ and 360 mV at 1.0 A cm^−2^) (Figure S8, Supporting Information), implying the autologous growth method greatly improves the OER activity. Note that the activity analysis of NF is excluded because of its exceptionally high overpotentials for OER. The LSV curves without iR correction were presented in Figure S9 (Supporting Information), which exhibit the similar results. In addition to high catalytic activity, CAPist‐S1 exhibits a lower Tafel slope of 32.2 mV dec^−1^ when compared with NiFe LDH (99.3 mV dec^−1^) and blank NF (208.7 mV dec^−1^) (Figure S10a, Supporting Information), indicating the OER proceeds via a faster kinetic route over CAPist‐S1. This observation is further supported by electrochemical impedance spectroscopy (EIS) measurements (Figure S10b, Supporting Information) and the corresponding equivalent circuit, where CAPist‐S1 has the smallest semicircle radius among all the samples, demonstrating its accelerated charge‐transfer kinetics. The double‐layer capacitance (*C*_dl_) estimated from cyclic voltammetry (CV) curves at different scan rates is used to calculate the corresponding electrochemical surface area (ECSA) (Figure S11a,b, Supporting Information). As shown in Figure S11c (Supporting Information), it is found that the *C*_dl_ value of CAPist‐S1 (7.17 mF cm^−2^) exceeds that of NiFe‐LDH (6.24 mF cm^−2^), demonstrating the exposure of more catalytic sites to the electrolyte for CAPist‐S1. Furthermore, in optical images (Figure S12, Supporting Information), the CAPist‐S1 electrode shows much fewer exposed metal surfaces and edges in its inner skeleton compared with NiFe LDH electrode, which indicates more uniform catalyst coverage over the whole CAPist‐S1 electrode. The more uniform catalyst coverage means the participation of more catalytic sites in the OER, consistent with *C*_dl_ measurement results. When the current density is normalized to ECSA (Figure S11d, Supporting Information) and loading mass (Figure S13, Supporting Information), CAPist‐S1 still has notably higher catalytic activity than NiFe‐LDH. Moreover, the turnover frequency (TOF) values of the prepared samples were also calculated by assuming all the metal atoms as active sites. As shown in Figure S14 (Supporting Information), the TOF values of CAPist‐S1 are significantly higher than those of NiFe LDH at various applied potentials in alkaline simulated seawater, highlighting the strong effect of this integrated electrode structure on the superior OER activity of CAPist‐S1.

The OER performance of the targeted CAPist‐S1 catalyst was subsequently measured in alkaline natural seawater. As shown in Figure 3b, the OER activities follow the order of CAPist‐S1 > NiFe‐LDH > NF, in line with the order in alkaline simulated seawater (The LSV curves measured in alkaline natural seawater without iR correction were presented in Figure S15, Supporting Information). The overpotentials to afford the current densities of 0.5 and 1.0 A cm^−2^ are only 193 and 220 mV for CAPist‐S1 in alkaline natural seawater, respectively, lower than the reference samples (Figure S16, Supporting Information) due to the favorable reaction kinetics (Figure S17a, Supporting Information) and fast charge transfer (Figure S17b, Supporting Information). The excellent OER activity of CAPist‐S1 toward alkaline natural seawater electrocatalysis can be further demonstrated by the high specific activity (Figure S18, Supporting Information), mass activity (Figure S19, Supporting Information) and TOF values (Figure S20, Supporting Information). Notably, the OER performance of CAPist‐S1 surpasses that of recently reported state‐of‐the‐art electrocatalysts in both alkaline simulated and natural seawater, as listed in Table S3 (Supporting Information).

The oxygen generation Faradaic efficiency of CAPist‐S1 was then measured in alkaline natural seawater via the drainage gas collection method. As shown in Figure 3d, CAPist‐S1 exhibits nearly 100% Faradaic efficiency under the current densities of 0.5 and 1.0 A cm^−2^, respectively, indicating its excellent OER selectivity. The post‐OER analysis of CAPist‐S1 in Figure S21 (Supporting Information) shows the preservation of surface nanosheet morphology and the crystalline NiFe LDH phase after OER catalysis, which reveals the good stability of CAPist‐S1. Furthermore, when CAPist‐S1 operated at an industrial‐level current density of 1.0 A cm^−2^ in alkaline natural seawater, an extraordinary long‐term stability over a period of 9000 h was achieved (Figure 3c). Figure 3e and Table S3 (Supporting Information) show the comparison of electrochemical stability for CAPist‐S1 with that for the recently reported OER electrocatalysts in alkaline simulated and natural seawater. The longest stability test of CAPist‐S1 at the industrial‐level current density of 1.0 A cm^−2^ suggests its bright prospects in practical seawater electrolysis. In addition, the Cl^−^ content in the electrolyte measured by ion chromatography at various time intervals during long‐term operation shows only slight fluctuation (Figure S22, Supporting Information), which further highlights the excellent OER stability and selectivity of CAPist‐S1 under the industrial‐level current density.

As shown in the inset of Figure 3c, it is evident that the electrochemical stability of NiFe LDH electrode suffers from rapid decline within a short period of 5 h. The corresponding SEM images and digital photograph of NiFe LDH after stability test depict obvious electrode deactivation and fracture (Figure S23, Supporting Information). In contrast, the morphology and structure of CAPist‐S1 remain intact after OER electrolysis (Figure S24, Supporting Information). To investigate the striking difference in the stability between NiFe LDH and CAPist‐S1, both electrodes (sized 1×2 cm^2^) were immersed in deionized water and subjected to ultrasonication. As presented in Figure S25a (Supporting Information), a more turbid solution is observed for NiFe LDH after ultrasonication, implying the easy exfoliation of catalyst layer from NF substrate. This phenomenon is further reflected by the corresponding SEM image, where only sparse nanosheets remain on the skeleton of the ultrasonication‐treated NiFe LDH electrode (Figure S25b, Supporting Information). Conversely, after ultrasonication, CAPist‐S1 still maintains a compact catalyst layer with nanosheet morphology on the NF surface (Figure S25c, Supporting Information). These results clearly reveal that the stronger catalyst‐support interaction in CAPist‐S1 contributes to its excellent long‐term stability during seawater electrolysis.

Owing to the similar chemical structures and compositions as well as surface nanosheet morphologies of the NiFe LDH and CAPist‐S1 electrodes, the disparity of their catalyst‐support interaction is probably a result of the differences in their bulk structures. To investigate this, a cross‐sectional SEM image of NiFe LDH was obtained to compare the bulk structures of the materials. In contrast to CAPist‐S1, in which a dense interlayer is generated between the NiFe LDH nanosheets and NF substrate (Figure 2h), the LDH nanosheets directly contact the metal substrate in the bulk structure of NiFe LDH (Figure S26a, Supporting Information). The dense NiFe LDH interlayer in CAPist‐S1 can strengthen the interaction between the nanosheet layer and NF substrate, resulting in high mechanical stability. Moreover, this dense interlayer is hypothesized to efficiently retard the penetration of Cl^−^ ions to NF surface and improve the corrosion resistance of the obtained CAPist‐S1 electrode. To corroborate the barrier effect of this dense interlayer, the residual Cl contents in the NiFe LDH and CAPist‐S1 electrodes after 2 h of electrolysis were subsequently measured via the ion chromatography analysis. It is found that the Cl content in the post‐OER NiFe LDH (19.87 ppm) exceeds that in the post‐OER CAPist‐S1 (6.93 ppm) by a factor of 2.9 (Figure S26b, Supporting Information), providing direct evidence that the dense interlayer can improve the resistance to Cl^−^ ions corrosion, thereby guaranteeing the stability of CAPist‐S1 electrode during seawater electrolysis. The high corrosion resistance of CAPist‐S1 can be further confirmed by the Tafel polarization curves,^[^ ^24^, ^44^ ^]^ where the corrosion potential of CAPist‐S1 (1.30 V) exhibits a notably positive shift when compared with that of NiFe LDH (1.25 V) (Figure S27, Supporting Information).

### 2.3 Deep Insights into the Stability of CAPist‐S1 Against Seawater Corrosion

In practice, Fe leaching is widely recognized as a major reason for the decreased electrochemical stability of NiFe LDH.^[^ ^22^, ^45^, ^46^ ^]^ Besides, Cl^−^ ions can bind on the metal sites of catalyst layer under anodic potentials, accelerating the dissolution of metal sites and leading to the poor stability.^[^ ^26^, ^42^ ^]^ As for CAPist‐S1, according to ICP measurements, the concentration of dissolved Fe in the alkaline simulated seawater electrolyte is determined to be 70.91 ppb after electrolysis under the current density of 0.5 A cm^−2^ for 6 h in an H‐type cell, indicating Fe leaching indeed occurs during OER electrolysis. Previous study revealed that balancing the rates of Fe dissolution and redeposition can establish dynamically stable active Fe sites, thereby improving the electrochemical stability of Fe‐modified hydro(oxy)oxides.^[^ ^47^ ^]^ In this regard, the long lifetime of CAPist‐S1 is also probably associated with the dynamically stable Fe besides the unique interlayer structure. However, considering the poor stability and rapid corrosion of NiFe LDH electrode during electrolysis, it can be reasonably concluded that the formation of the dynamically stable Fe in CAPist‐S1 is closely related to the dense NiFe LDH interlayer.

**Figure 4:**
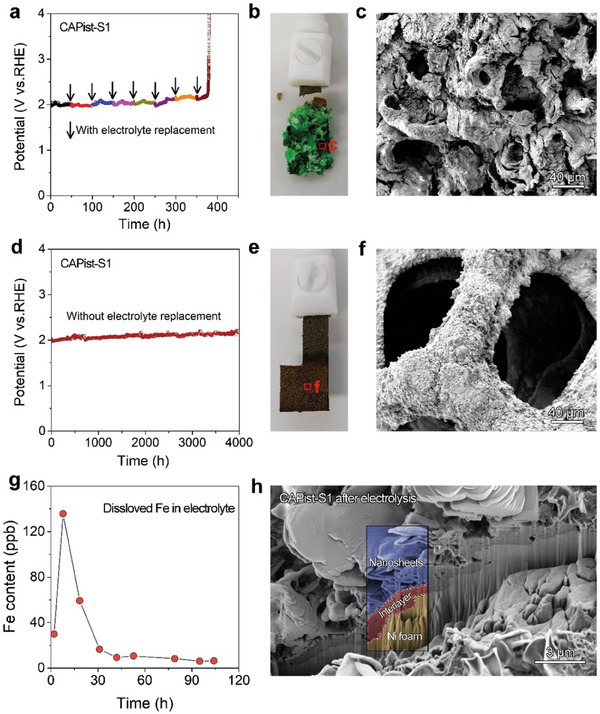
Stability tests of CAPist‐S1 a) with electrolyte replacement at intervals of 50 h and d) without electrolyte replacement in alkaline simulated seawater. Digital photographs of CAPist‐S1 after the stability tests b) with and e) without electrolyte replacement. SEM images of CAPist‐S1 skeleton after the stability tests c) with and f) without electrolyte replacement. g) Content of dissolved Fe in electrolyte as a function of electrolysis time. h) Cross‐sectional SEM image of CAPist‐S1 after 400 h of electrolysis obtained using focused‐ion‐beam (FIB) milling.

In order to disclose the relationship between the dynamically stable Fe and NiFe LDH interlayer and thoroughly understand the excellent stability of CAPist‐S1, the electrolysis experiments with and without electrolyte replacement were carried out. In the electrolyte replacement experiment, fresh alkaline simulated seawater was applied at intervals of 50 h, and a noticeable potential increase and electrode corrosion were observed for CAPist‐S1 after seven cycles of this process (≈370 h) (**Figure** **4**a–c), indicating the gradual deterioration of the protective function for the dense NiFe LDH interlayer during electrolyte replacement. After the first electrolyte replacement and then 6 h of electrolysis, the dissolved Fe content of 69.37 ppb is detected in the fresh electrolyte. This result reveals that although the dense interlayer plays a crucial role in the superior resistance to Cl^−^ ions corrosion, the structural stability of this dense interlayer can be gradually impaired by continuous Fe leaching during electrolyte replacement, which finally causes corrosion of the CAPist‐S1 electrode. Herein, it is anticipated that the lifetime of CAPist‐S1 electrode during electrolyte replacement can be prolonged by adding tiny amount of Fe ions in the fresh electrolyte (Figure S28, Supporting Information).

Without electrolyte replacement, the CAPist‐S1 can function stably for more than 4000 h (Figure 4d), and no electrode corrosion is observed (Figure 4e,f), suggesting that the dissolved Fe makes a significant contribution to the electrochemical stability of CAPist‐S1. Moreover, the content of Fe dissolved in electrolyte undergoes three distinct stages as a function of electrolysis time: a rapid increase at the initial electrolysis process, followed by a stepwise decrease, and finally reaches a constant level (Figure 4g and Table S4, Supporting Information). The negligible dissolution rate of Fe in the third stage indicates the further leaching of Fe is inhibited during prolonged electrolysis, consequently ensuring the structural stability of the dense NiFe LDH interlayer in CAPist‐S1 (Figure 4h; Figure S29, Supporting Information). Based on these results, the dissolved Fe species are confirmed to be associated with the electrochemical stability of CAPist‐S1 during OER.

In principle, the dynamically stable Fe species can also form over the NiFe LDH electrode. However, the absence of the dense interlayer leads to the rapid corrosion of NF substrate by Cl^−^ ions, thus the breakage of NiFe LDH electrode occurred before the formation of these dynamically stable Fe species. This result confirms that the dense interlayer in the prepared catalyst is a significant prerequisite for further investigating its surface chemistry during prolonged OER reaction. As a consequence, the relationship between the dynamically stable Fe and NiFe LDH interlayer in CAPist‐S1 can be depicted as following: the dense NiFe LDH interlayer as a blocking layer against the penetration of Cl^−^ ions onto NF substrate enables stable electrode structure, thereby ensuring sufficient time for the formation of dynamically stable Fe species to counter further Cl^−^ attack on catalyst layers.

**Figure 5:**
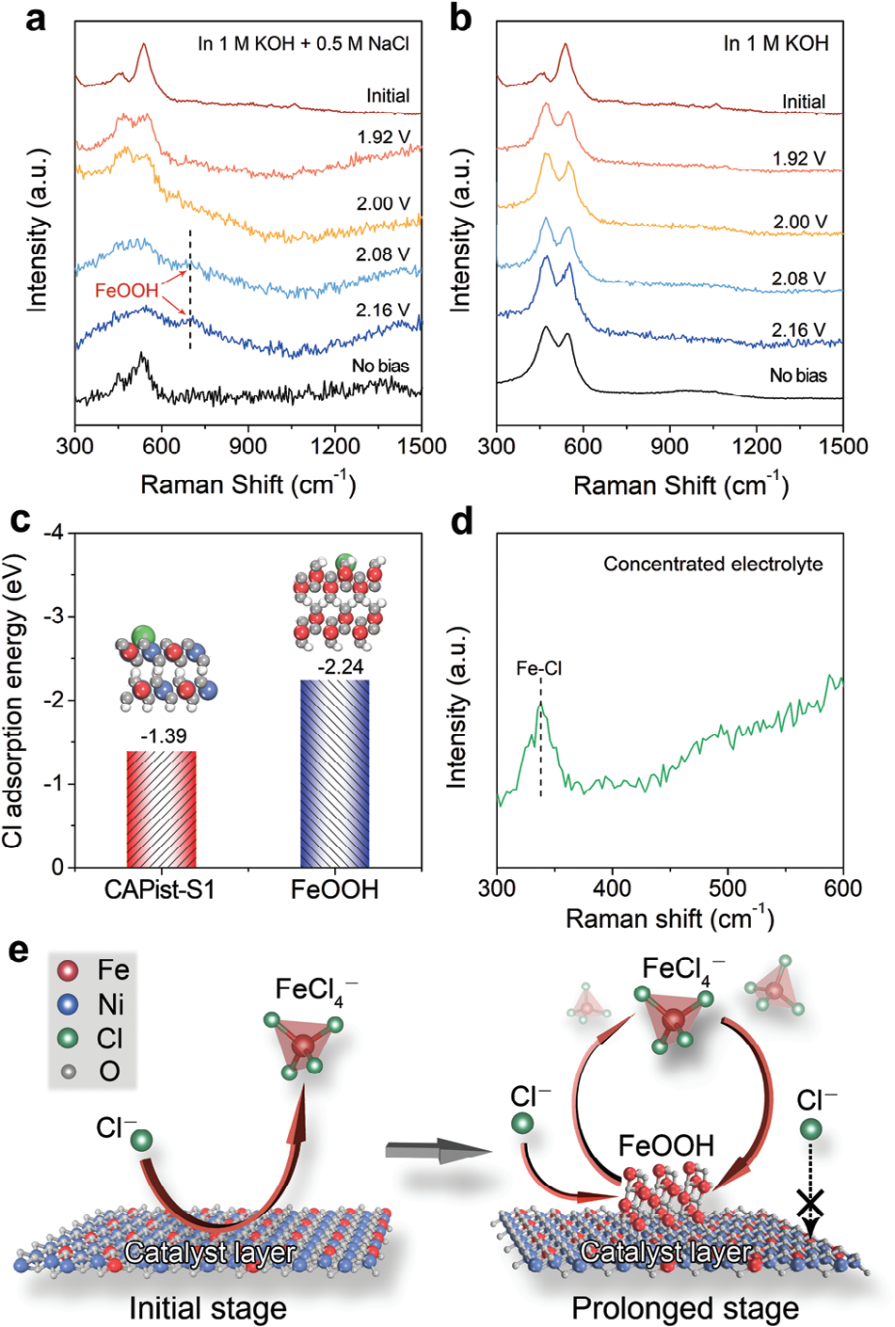
In situ Raman spectra of CAPist‐S1 in a) alkaline simulated seawater and b) 1 M KOH under various potentials. c) Adsorption energy of Cl^−^ on CAPist‐S1 and FeOOH. Inset shows the structure models of Cl‐adsorbed CAPist‐S1 and Cl‐adsorbed FeOOH. The blue, red, grey, and white balls represent Ni, Fe, O and H atoms, respectively. d) Raman spectra of the concentrated electrolyte collected from CAPist‐S1 after electrolysis for 6 h. e) Schematic diagram of the proposed catalyst protection mechanism.

To probe the surface change of CAPist‐S1 during OER, the in situ Raman spectroscopy was subsequently recorded in alkaline simulated seawater. The two characteristic peaks appearing at 469.1 and 546.3 cm^−1^ (**Figure** **5**a) within the potential range of 1.92–2.16 V can be attributed to the Ni‐O vibrations in NiOOH.^[^ ^48^, ^49^ ^]^ Herein, the decreased intensity of NiOOH peaks as the potential increases can be attributed to the prolonged residence time of formed oxygen bubbles on catalyst surface caused by the presence of Cl^−^ ions (Figure S30, Supporting Information). In addition, a broad peak at 698.5 cm^−1^ associated with FeOOH emerges as the potential reaches 2.08 V.^[^ ^43^, ^50^ ^]^ However, when the applied potential is removed, the characteristic peak of FeOOH completely disappears, indicating the in‐situ formation of FeOOH. Previous research has demonstrated that the FeOOH in the NiFe LDH/FeOOH heterostructure efficiently prevents Cl^−^ ions from eroding NiFe LDH.^[^ ^51^ ^]^ Thus, it is reasonable to assume that the in situ generated FeOOH interacts with CAPist‐S1 and acts as a protective media impeding Cl^−^ attack. As demonstrated in density functional theory (DFT) calculations (Figure 5c), the Cl^−^ ions preferentially adsorb on the FeOOH surface instead of the CAPist‐S1 surface due to the lower adsorption energy. Therefore, the FeOOH layer covering the CAPist‐S1 surface can protect against Cl^−^ attack on the metal sites of catalyst layer, ensuring the structural stability of the dense NiFe LDH interlayer. Notably, when the in situ Raman measurements were performed in 1 M KOH (without Cl^−^), the peaks associated with NiOOH remained observable through all the applied potentials, while no FeOOH‐related characteristic peaks appeared during this process (Figure 5b). This result excludes the direct FeOOH formation from CAPist‐S1 surface and clearly emphasizes the indispensable role of Cl^−^ ions in promoting the in‐situ generation of the FeOOH.

In the presence of Cl^−^ ions, metal atoms are generally leached from catalysts or substrates in the form of MCl_x_^−^.^[^ ^52^ ^]^ In this regard, the FeCl_4_^−^ ions generated in the electrolyte during electrolysis are presumably responsible for the in‐situ generation of FeOOH. To confirm this, after electrolysis over CAPist‐S1, the electrolyte was collected and subsequently freeze‐dried to remove the solvent for the Raman measurements. As shown in Figure 5d, the Fe‐Cl vibration peak at 338 cm^−1^ provides solid evidence to the existence of FeCl_4_^−^ ions in the electrolyte,^[^ ^53^ ^]^ which serve as precursors for the in‐situ formation of FeOOH.

Based on these results, we propose a mechanism for the excellent stability of CAPist‐S1 (Figure 5e). During the initial electrolysis stage (Figure 4c), due to the blocking effect of the dense NiFe LDH interlayer, the Cl^−^ ions in electrolyte are first adsorbed on the positively polarized CAPist‐S1 surface rather than penetrating to the NF substrate, thus preventing rapid corrosion of the CAPist‐S1. Then, the remaining free Cl^−^ ions in electrolyte continuously coordinate with the Cl‐adsorbed Fe sites, leading to the leaching of these metal sites from CAPist‐S1 to the electrolyte in the form of FeCl_4_^−^. This explains the rapid increase of Fe content in electrolyte during the initial electrolysis stage. The produced FeCl_4_^−^ ions subsequently participate in the formation of the FeOOH, resulting in the decrease of the total Fe content in electrolyte. Due to the more favorable adsorption for Cl^−^ ions, the Fe sites in FeOOH will become the dominating adsorption sites of Cl^−^ ions. As electrolysis continues, a dynamic equilibrium between Fe dissolution and redeposition over FeOOH is then established: FeCl_4_^−^ + 3OH^−^ ↔ FeOOH + H_2_O + 4Cl^−^, which contributes to the final negligible dissolution rate of Fe in electrolyte. Overall, the dense NiFe LDH interlayer guarantees electrode stability in the initial electrolysis stage, allowing the establishment of a dynamic equilibrium between Fe leaching and redeposition over the in situ formed FeOOH. This dynamic equilibrium can in turn inhibit the further leaching of Fe from the catalyst layer, ensuring the structural stability of the dense NiFe LDH interlayer.

### 2.4 Industrial Current Density AEM Seawater Electrolysis with CAPist‐S1 as Anode

**Figure 6:**
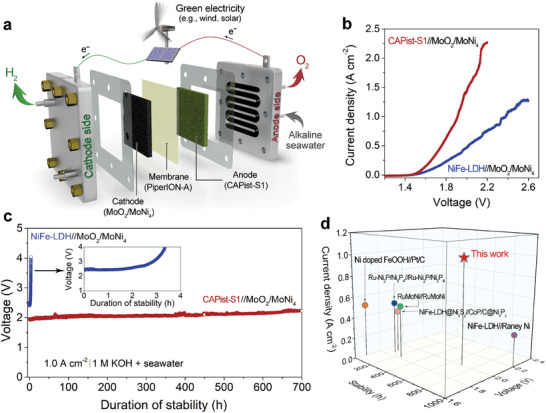
a) Schematic diagram of the AEM seawater electrolyzer. b) LSV curves of AEM water electrolyzers for the CAPist‐S1//MoO_2_/MoNi_4_ and NiFe LDH//MoO_2_/MoNi_4_ cells without iR compensation at room temperature using alkaline natural seawater as electrolyte. c) Chronopotentiometric measurements of AEM seawater electrolyzers for the CAPist‐S1//MoO_2_/MoNi_4_ and NiFe LDH//MoO_2_/MoNi_4_ cells at the current density of 1.0 A cm^−2^. The inset shows the enlarged chronopotentiometric curve of NiFe LDH//MoO_2_/MoNi_4_ cell. d) Summary of recently reported AEM electrolyzers toward seawater electrolysis.^[^ ^42^, ^54^, ^55^, ^56^, ^57^ ^]^ All the measurements were tested by using alkaline seawater as a circulated electrolyte. Detailed information can be found in Table S5 (Supporting Information).

Motivated by the excellent OER performance of CAPist‐S1, an alkaline seawater AEM electrolyzer was assembled by using CAPist‐S1 as the anode, NF supported MoO_2_/MoNi_4_ as the cathode, and commercial PiperION‐A as the anion exchange membrane (**Figure** **6**a). The AEM electrolyzer with NiFe LDH as the anode was also prepared for comparison. Before the measurement, the surface of Ni end plate at anode side was covered with the NiFe LDH through the same method as that for CAPist‐S1 (Figure S31, Supporting Information), which serves as a protective layer to avoid the Cl^−^ corrosion. As shown in Figure 6b, the AEM electrolyzer assembled from CAPist‐S1//MoO_2_/MoNi_4_ delivers a current density of 1.0 A cm^−2^ at the cell voltage of 1.91 V under room temperature, ≈470 mV lower than that of the AEM electrolyzer assembled from NiFe LDH//MoO_2_/MoNi_4_. The long‐term stability of the AEM electrolyzer assembled from CAPist‐S1//MoO_2_/MoNi_4_ was demonstrated in the chronopotentiometry (CP) curve (Figure 6c), where negligible potential fluctuations were observed at the large current density of 1.0 A cm^−2^ over 700 h. In contrast, the AEM electrolyzer assembled from NiFe LDH//MoO_2_/MoNi_4_ displays extremely poor stability under such high current density due to the corrosion of NiFe LDH anode, as presented in the digital photographs (Figure S32, Supporting Information). The outstanding long‐term stability of CAPist‐S1 reflected in the AEM seawater electrolyzer further highlights its promising application prospects. To the best of our knowledge, the stability of the CAPist‐S1 anode‐based AEM electrolyzer for seawater electrolysis reaches a record setting at the industrial‐level current density of 1.0 A cm^−2^ (Figure 6d and Table S5, Supporting Information).

## 3 Conclusion 

In summary, we developed a corrosion‐resistant NiFe LDH electrode (CAPist‐S1) with interlayer structure for highly efficient and stable alkaline seawater oxidation. The resulting CAPist‐S1 exhibits ultralow overpotentials of 200 and 220 mV to launch an industrial‐level current density of 1.0 A cm^−2^ in alkaline simulated and natural seawater, respectively, as well as extraordinary long‐term stability over 9000 h at 1.0 A cm^−2^ in alkaline natural seawater. When an AEM seawater electrolyzer was assembled with CAPist‐S1 as the anode, excellent durability for over 700 h was achieved at 1.0 A cm^−2^ at room temperature. The outstanding performance of CAPist‐S1 can be ascribed to the presence of a dense NiFe LDH interlayer in its bulk structure. This dense interlayer not only enhances the catalyst‐support interaction but also efficiently retards the diffusion and penetration of Cl^−^ to substrate surface. Further experimental analysis reveals that the dense interlayer is critical for establishing a dynamic equilibrium between Fe leaching and redeposition over the in situ formed FeOOH, while this dynamic equilibrium can in turn stabilize the structure of the dense NiFe LDH interlayer during prolonged OER electrolysis. These results provide new insight into the rational design of highly efficient, stable, and large‐scale electrocatalysts for sustainable hydrogen production from seawater splitting.

## Conflict of Interest

The authors declare no conflict of interest.

## Supporting information



Supporting Information

## Data Availability

The data that support the findings of this study are available in the supplementary material of this article.
