# Supplementary material for: Anion Exchange Membrane Seawater Electrolysis at 1.0 A cm−2 With an Anode Catalyst Stable for 9000 H
Source: Adv Sci (Weinh). 2025 Mar 28;12(22):2416661. doi: 10.1002/advs.202416661 (PMC12165096; doi:10.1002/advs.202416661)
Supplement: Supplementary file 1 — Supporting Information [file ADVS-12-2416661-s001.docx]

Supporting Information

Anion Exchange Membrane Seawater Electrolysis at 1.0 A cm^-2^ with an Anode Catalyst Stable for 9000 hours

Jian Du^+^, Zhiheng Li^+^, Linqin Wang, Yunxuan Ding, Wentao Ye, Wenxing Yang and Licheng Sun*

**Materials**

Nickel nitrate hexahydrate (Ni(NO_3_)_2_·6H_2_O, 99%), ferrous sulfate heptahydrate (FeSO_4_·7H_2_O, 99%), urea (CO(NH_2_)_2_, 99%), sodium chloride (NaCl, 99.999%), 2-propanol (C_3_H_7_OH, 99.9%) and ethylene glycol (C_2_H_4_(OH)_2_, 99.5%) were purchased from Adamas-Beta. Iron nitrate nonahydrate (Fe(NO_3_)_3_·9H_2_O, 99.9%) and potassium hydroxide (KOH, 95%) were obtained from Aladdin chemical company. Ammonium fluoride (NH_4_F, 98%) was purchased from Sigma-Aldrich. PiperION-A membrane was purchased from Shanghai ZaiAng Material Technology Co., ltd. The seawater was collected from Mariana Trench. Nickel foam (thickness: 1 mm, bulk density: 2000 mg cm^-3^) was purchased from commercial supplier.

**Synthesis of CAPist-S1 electrode**

The CAPist-S1 electrode was prepared based on the previously work of our group but with minor modification.^[1]^ The difference lies in the added Fe content, which was increased in this manuscript due to its critical role in the sustained stability. Typically, 1.02 g Ni(NO_3_)_2_·6H_2_O (3.5 mmol) was firstly dissolved in 2-propanol (15 mL) to form a clear solution. The aqueous solution (5 mL) containing 0.33 g FeSO_4_·7H_2_O (1.2 mmol) was then added dropwise into the above solution under stirring. Thereafter, a piece of acid-treated nickel foam (NF, 1.5 × 3.0 cm^2^) was vertically deposited in the mixed solution at room temperature for 24 h. The as-prepared electrode was subsequently washed with deionized water several times and dried under vacuum condition overnight to give the final target catalyst.

**Synthesis of NiFe LDH electrode**

The NiFe LDH electrode was fabricated through a typical solvothermal method^[2]^. Generally, 0.44 g Ni(NO_3_)_2_·6H_2_O (1.5 mmol), 0.20 g Fe(NO_3_)_3_·9H_2_O (0.5 mmol), 0.72 g urea (12 mmol) and 0.29 g NH_4_F (8 mmol) were dissolved in deionized water (20 mL) and glycol (10 mL) to form a clear mixed solution. After that, the obtained solution together with a piece of acid-treated NF (1.5×3.0 cm^2^) were transferred into a 50 mL Teflon-lined autoclave and heated at 120 ℃ for 12 h. After naturally cooling to room temperature, the as-prepared electrode was subsequently washed with deionized water several times, then dried under vacuum condition overnight to give the final NiFe LDH electrode.

**Materials characterization**

The morphology and structure of the as-prepared catalysts were investigated by Analytical Field Emission Scanning Electron Microscope (Gemini 450), Focused Ion Beam Scanning Electron Microscope (Helious 5 UX) and High-Resolution Transmission Electron Microscope (Talos F200X G2). The powder X-ray diffractometer (D8 Advance) with Cu Kα radiation was used to analyze the crystal structures of the catalysts. The elemental composition and valence states in the catalysts were determined by X-ray photoelectron spectroscopy (ESCALAB Xi+). The binding energy (BE) was calibrated with respect to the C 1s peak at 284.8 eV. The *ex situ* and *in situ* Raman spectra were collected on a Raman spectrometer (Alpha300R). For the *in situ* Raman measurements, the chronoamperometric measurements at various applied potentials (1.92, 2.0, 2.08 and 2.16 V) were conducted in alkaline natural seawater under room temperature by using a home-made cell for 3 mins. Since the relatively higher electrochemical resistance in the home-made cell when compared with that in the three-electrode setup (vide infra), the current densities range from 10~100 mA cm^-2^ under the selected potentials. The metal contents in the samples were quantitatively analyzed by Inductively coupled plasma mass spectrometer (iCAP RQ). The Cl content in electrolyte was calculated by Ion chromatography (ICS 6000).

**Electrochemical measurements**

Electrochemical measurements were performed on the Autolab electrochemical workstation (PGSTAT 302N) connected with a current booster (10 A) in a three-electrode setup with the as-prepared catalyst as the working electrode, Pt mesh as the counter electrode and Hg/HgO as the reference electrode at room temperature using alkaline simulated (1 M KOH + 0.5 M NaCl) and natural (1 M KOH + seawater) seawater as electrolyte, respectively. All the potentials were converted to reversible hydrogen electrode (RHE) scale, E_RHE_ = E_Hg/HgO_ + 0.0592pH + 0.12 V.

The OER activity was determined by using linear sweep voltammetry (LSV) with manual *iR* compensation at a scan rate of 5 mV s^-1^ under room temperature. The Tafel plots tests were carried out to evaluate the reaction kinetics of the catalysts and the corresponding Tafel slopes were calculated from LSV curves by plotting overpotential against log (current density). The electrochemical impedance spectroscopy (EIS) of catalysts were measured under the potential of 1.52 V in the frequency range from 0.01 to 10^5^ HZ with an amplitude of 5 mV. According to the EIS measurements, the *iR*-corrected LSV curves can be obtained by the following equation: E = E_0_ - iR_s_, where R_s_ represents the solution resistance. The electrochemically active surface area (ECSA) estimated from the double-layer capacitance (C_dl_) was calculated by performing CV tests in the non-faradic region from 0.2 to 0.3 V vs. Hg/HgO at scan rates of 20, 40, 60, 80, 100 and 120 mV s^-1^, respectively. The corrosion resistance properties of the catatlysts were evalutated by the Tafel plots testin the potential range from 0.9 V to 1.5 V at a scan rate of 1 mV s^-1^. The stability was evaluated by conducting chronopotentiometric measurement at a constant density of 1.0 A cm^-2^ in alkaline natural seawater under room temperature.

The OER faradic efficiency of the catalyst was determined by a classical drainage gas collection method in the three-electrode model with the use of alkaline natural seawater as electrolyte. The electrolysis was performed under constant current densities of 0.5 and 1.0 A cm^-2^ for 1h, and the actual amount of O_2_ produced at every 10 mins was recorded. The Faradic efficiency was calculated from the total amount of charge passed through the cell (Q) and the total amount of oxygen produced (n_O2_) according to the equation, Faradaic efficiency (FE) = 4F × n_O2_/Q, where F is the Faraday constant (96485 C mol^-1^).

**The fabrication and performance tests of anion exchange membrane (AEM) seawater electrolyzer**

The AEM seawater electrolyzer was fabricated by the direct assembly of OER catalyst, HER catalyst, two gas diffusion layers (NF, 1 × 1 cm^2^), AEM and two nickel end plates with triple serpentine channels. Herein, the CAPist-S1 was used as the anode, the NF supported Ni_4_Mo/MoO_2_ (NF-Ni_4_Mo/MoO_2_) was selected as the cathode^[3]^ and the commercial PiperION-A (thickness: 40 μm) membrane was employed as the AEM. The PiperION-A membrane was then sandwiched between the CAPist-S1 anode and NF-Ni_4_Mo/MoO_2_ cathode to prepare membrane electrode assembly (MEA), which was subsequently integrated with nickel end plates into AEM electrolyzer.

The performance of the constructed AEM seawater electrolyzer was evaluated in flowing alkaline natural seawater solution under room temperature. The LSV curves without *iR* compensation were tested in the potential range of 1.0~2.6 V at a scan rate of 5 mV s^-1^. The long-term durability of the AEM seawater electrolyzer was performed at a constant current density of 1.0 A cm^-2^.

**Theoretical computation details**

All DFT calculations in the work were carried out using the Vienna *ab initio* simulation program (VASP)^[4,5]^. The projector-augmented wave (PAW) method was utilized to describe the pseudopotential^[5,6]^, and the plane-wave basis expansion cut-off energy was set to be 450 eV. For exchange and to correlate the functional, the generalized gradient approximation (GGA) was used with Perdew–Burke–Ernzerhof (PBE) to perform all spin-polarized calculations^[7]^. The NiFeOOH (001) and FeOOH (001) surfaces were built with a *p*(2 × 2) supercell. A ~15 Å vacuum layer was used to eliminate the interaction between neighboring slabs. A 2 × 2 × 1 Monkhorst–Pack k-point mesh sampling was used for all optimizations. The equilibrium was reached when the forces on the relaxed atoms and the energies in the self-consistent iterations became less than 0.05 eV/Å and 10^-5^ eV, respectively. The van der Waal (vdW) interaction was described by the DFT-D3 method^[8,9]^.

The Gibbs free energy (ΔG) can be expressed as

$\Delta G=\Delta E+\Delta ZPE-T\Delta S-eU+k_{B}TIn(H^{+})$ (1)

where ΔE is the difference of the total energy between the reactants and products. ΔZPE and TΔS are the thermodynamic corrections of zero-point-energy (ZPE) and entropy (S) derived from vibrational partition function at 300 K. The *e* is the transferred electron and the *U* is the external potential, while k_B_TIn(H^+^) term is the free energy correction of pH. Here, all thermodynamic corrections were carried out under standard conditions. The overpotential (𝜂) can be determined by

$\eta=\Delta G/e-1.23 V$ (2)

**References**

[1] Z. Li, G. Lin, L. Wang, H. Lee, J. Du, T. Tang, G. Ding, R. Ren, W. Li, X. Cao, S. Ding, W. Ye, W. Yang, L. Sun, *Nat. Catal*. **2024**, *7*, 944.

[2] H. Liu, W. Shen, H. Jin, J. Xu, P. Xi, J. Dong, Y. Zheng, S.-Z. Qiao, *Angew. Chem., Int. Ed*. **2023**, *62*, e202311674.

[3] J. Zhang, T. Wang, P. Liu, Z. Liao, S. Liu, X. Zhuang, M. Chen, E. Zschech, X. Feng, *Nat. Commun*, **2017**, *8*, 15437.

[4] G. Kresse, J. Furthmüller, *Phys. Rev. B* **1996**, *54*, 11169.

[5] G. Kresse, D. Joubert, *Phys. Rev. B* **1999**, *59*, 1758.

[6] P. E. Blöchl, *Phys. Rev. B* **1994**, *50*, 17953.

[7] J. P. Perdew, K. Burke, M. Ernzerhof, *Phys. Rev. Lett.* **1996**, *77*, 3865.

[8] S. Grimme, J. Antony, S. Ehrlich, H. Krieg, *J. Chem. Phys*. **2010**, *132*, 154104.

[9] S. Grimme, S. Ehrlich, L. Goerigk, *J. Comput. Chem*. **2011**, *32*, 1456.


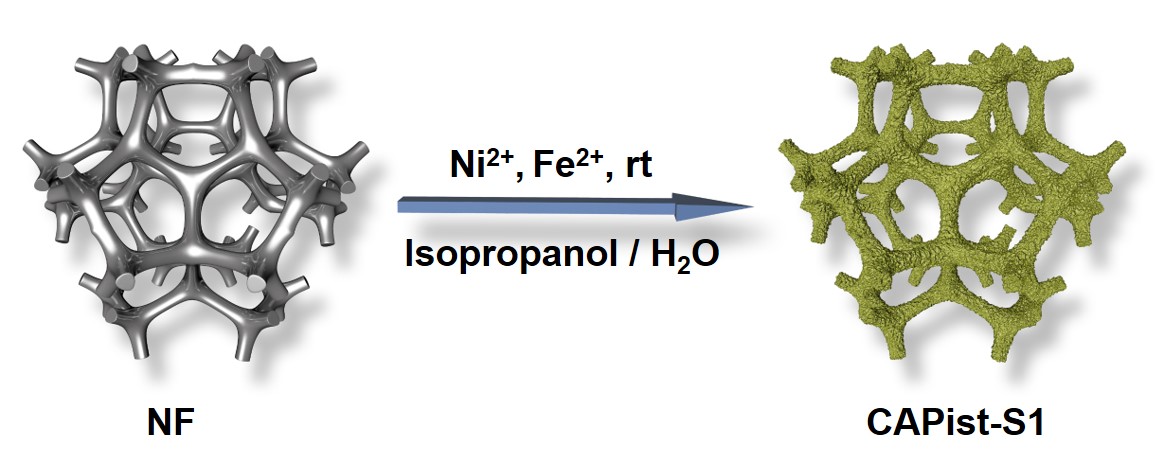


**Figure S1**. The synthetic process of CAPist-S1.


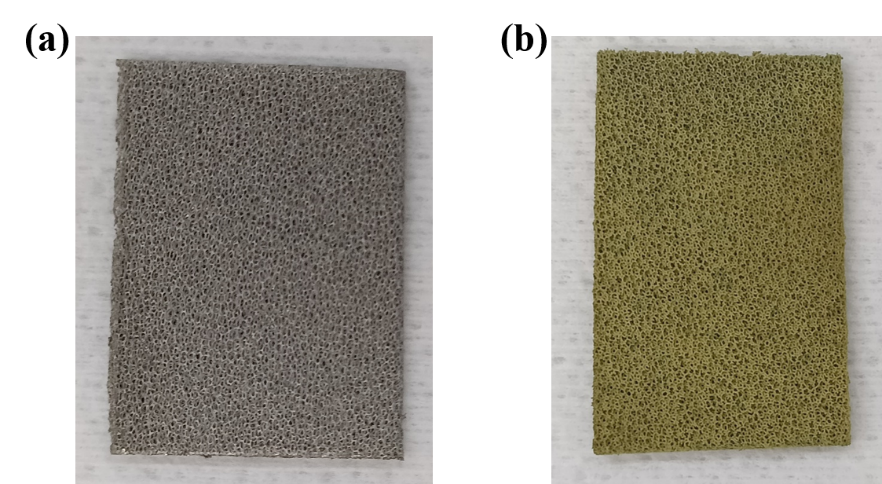


**Figure S2**. The digital photographs of (a) NF blank and (b) CAPist-S1.


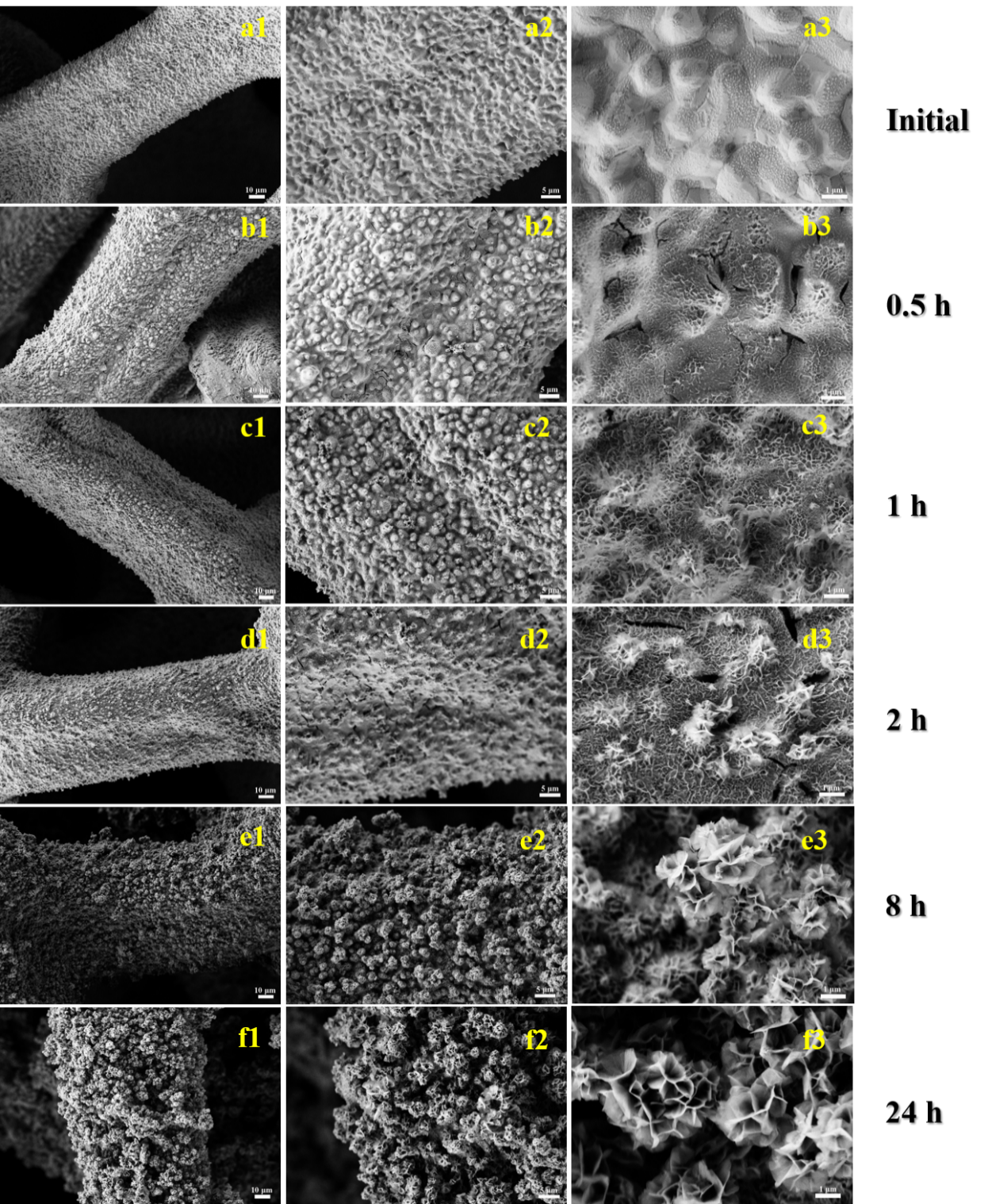


**Figure S3**. (a1-a3) SEM images of NF blank. SEM images of CAPist-S1 obtained with various catalyst growth time of (b1-b3) 0.5 h, (c1-c3) 1 h, (d1-d3) 2 h, (e1-e3) 8 h and (f1-f3) 24 h under different magnifications.


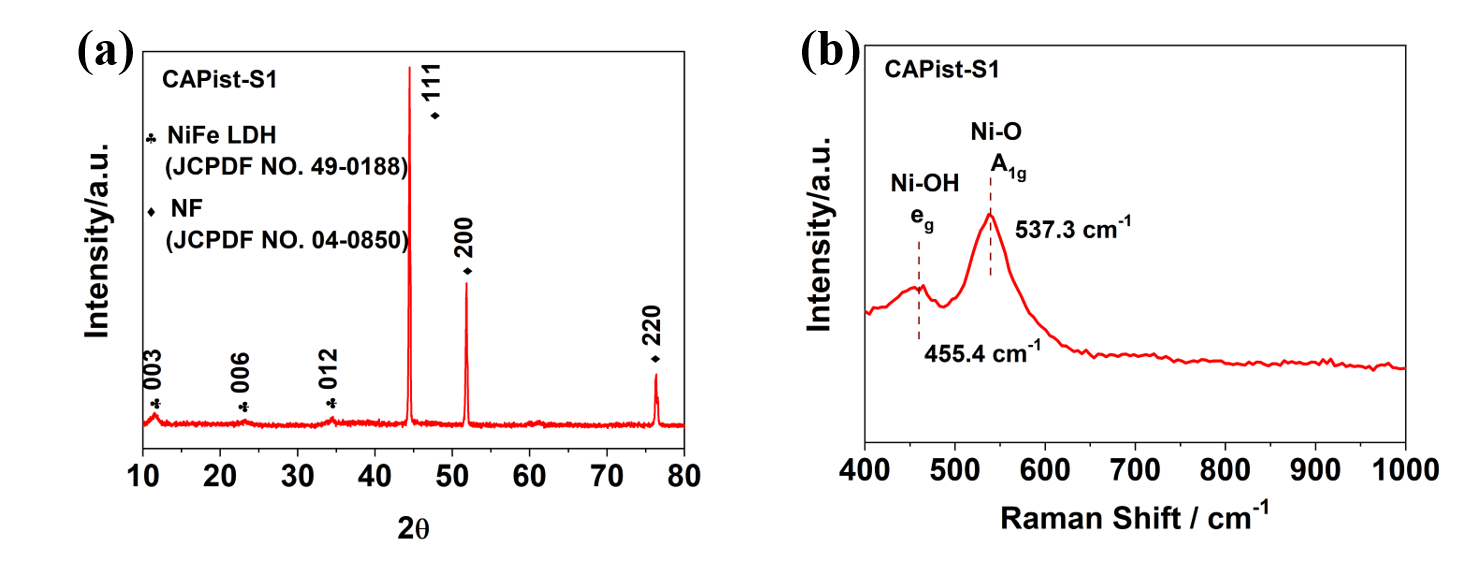


**Figure S4.** (a) XRD pattern and (b) Raman spectrum of CAPist-S1.


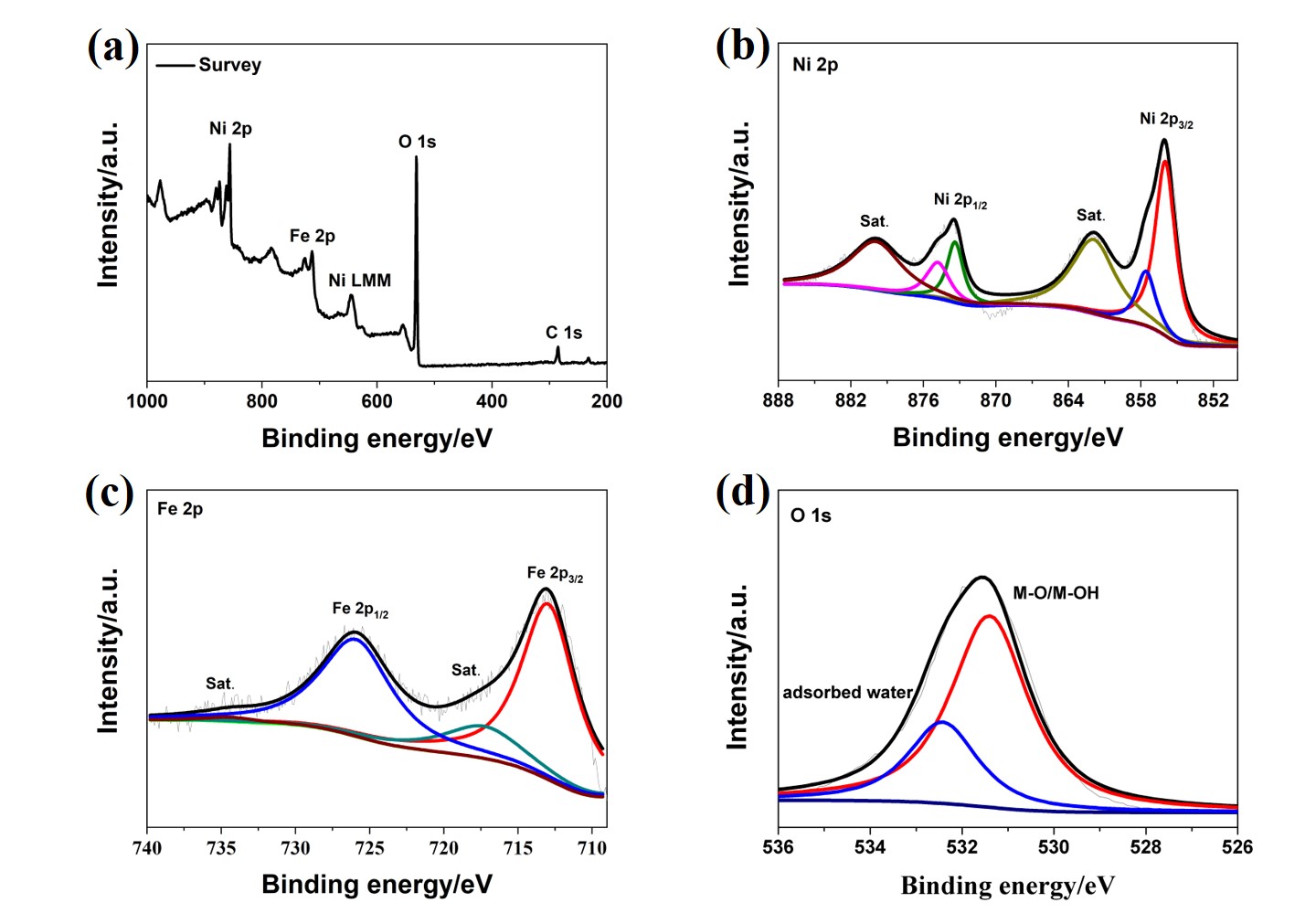


**Figure S5**. (a) The XPS survey spectrum of CAPist-S1. High-resolution XPS spectra of (b) Ni 2p, (c) Fe 2p and (d) O 1s of CAPist-S1.


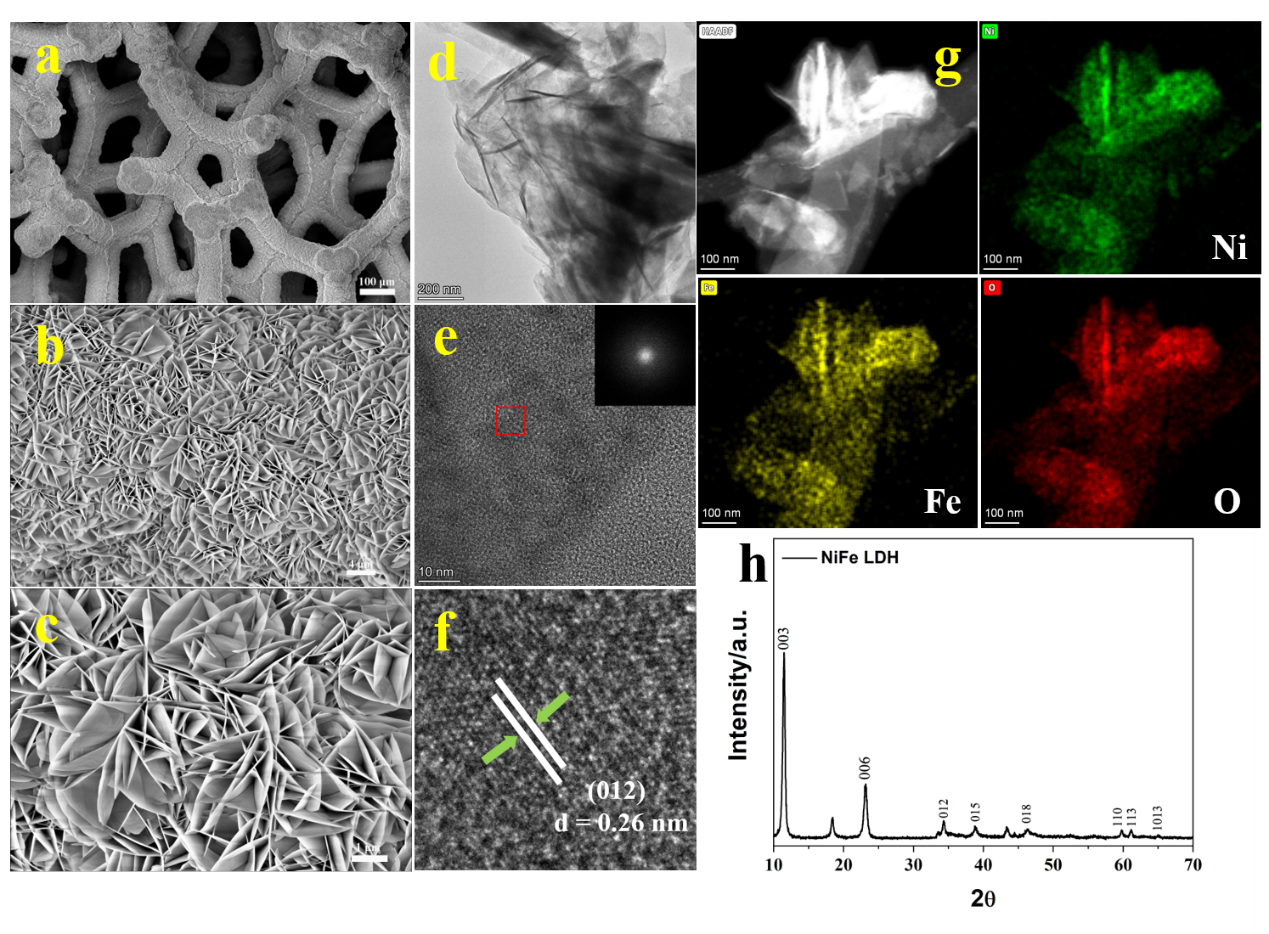


**Figure S6**. (a-c) SEM images of NiFe LDH under different magnifications. (d) TEM and (e) HRTEM images of NiFe LDH (inset shows the SAED pattern in the red frame region). (f) HRTEM image of the red frame region in Figure S6e. (g) HADDF-STEM image and Ni map, Fe map and O map for NiFe LDH. (h) XRD pattern of NiFe LDH powder. The NiFe LDH powder was collected through the ultrasonication treatment of NiFe LDH electrode, thus avoiding the overshadowing of NF substrate during XRD test.


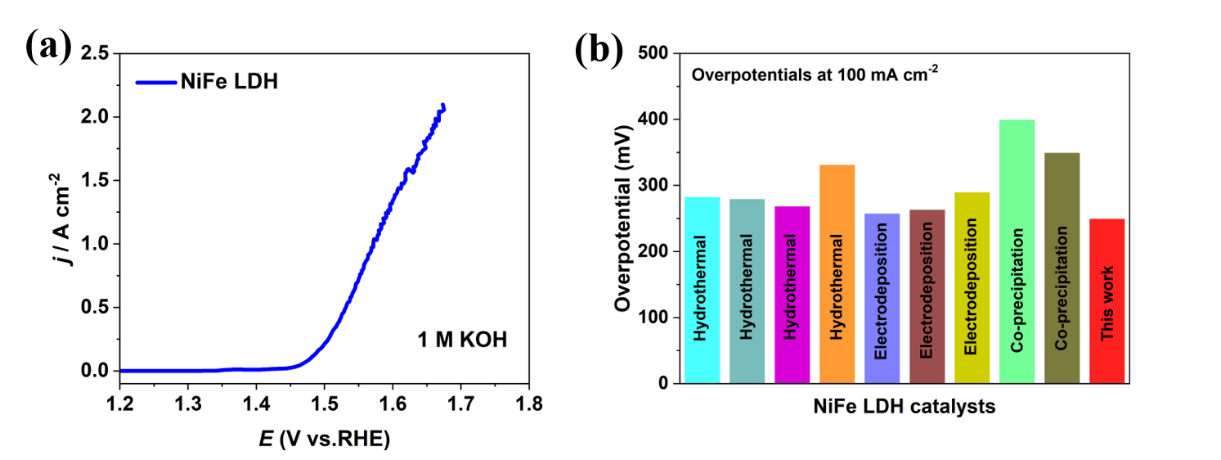


**Figure S7**. (a) LSV curve of NiFe LDH measured in 1 M KOH. (b) Comparison of overpotentials at 100 mA cm^-2^ in 1 M KOH for NiFe LDH and other recently reported NiFe LDH electrodes synthesized from various strategies. Detailed information can be found in Table S2.


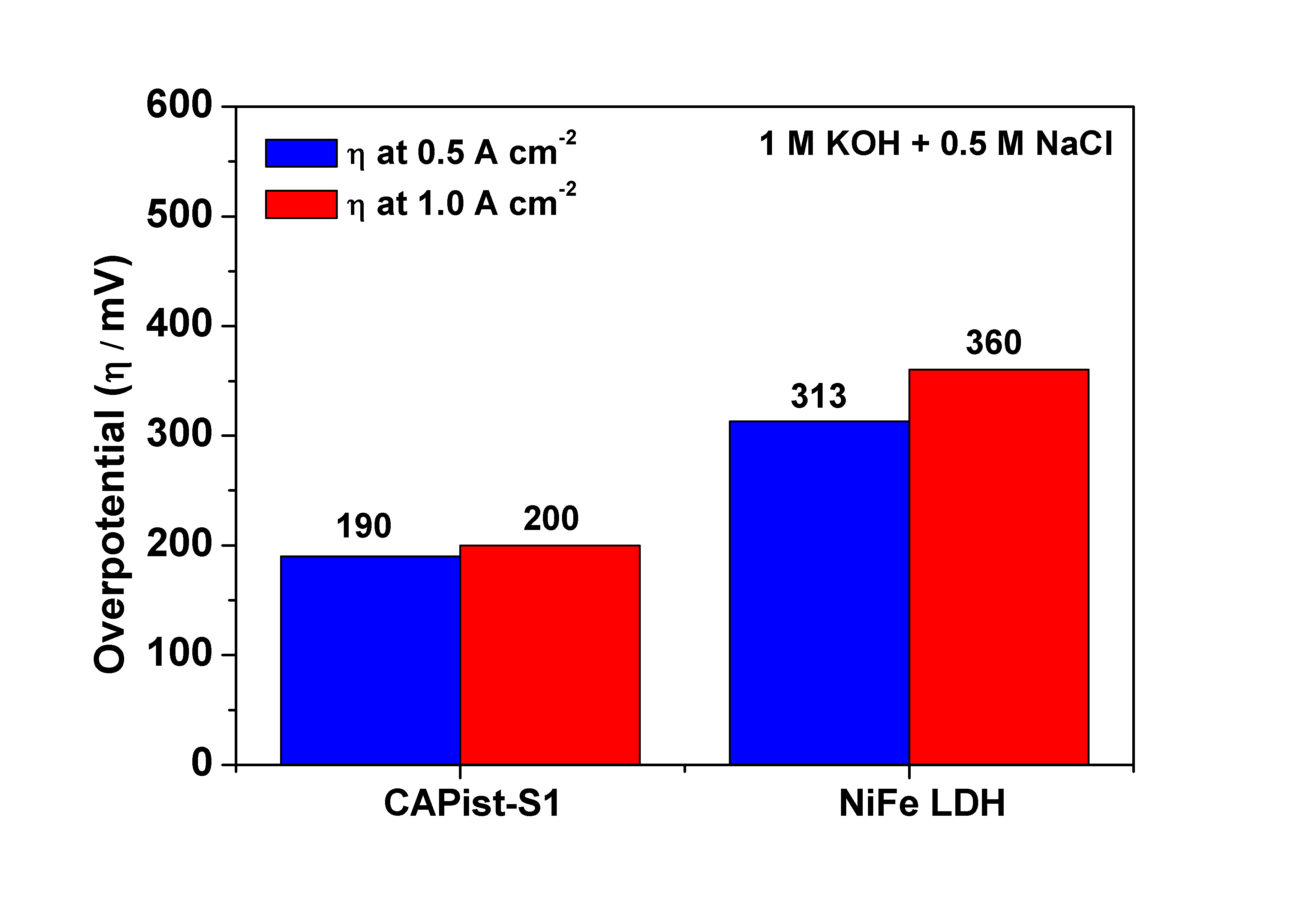


**Figure S8**. Comparison of overpotentials for CAPist-S1 and NiFe LDH at the current densities of 0.5 and 1.0 A cm^-2^ in alkaline simulated seawater, respectively.


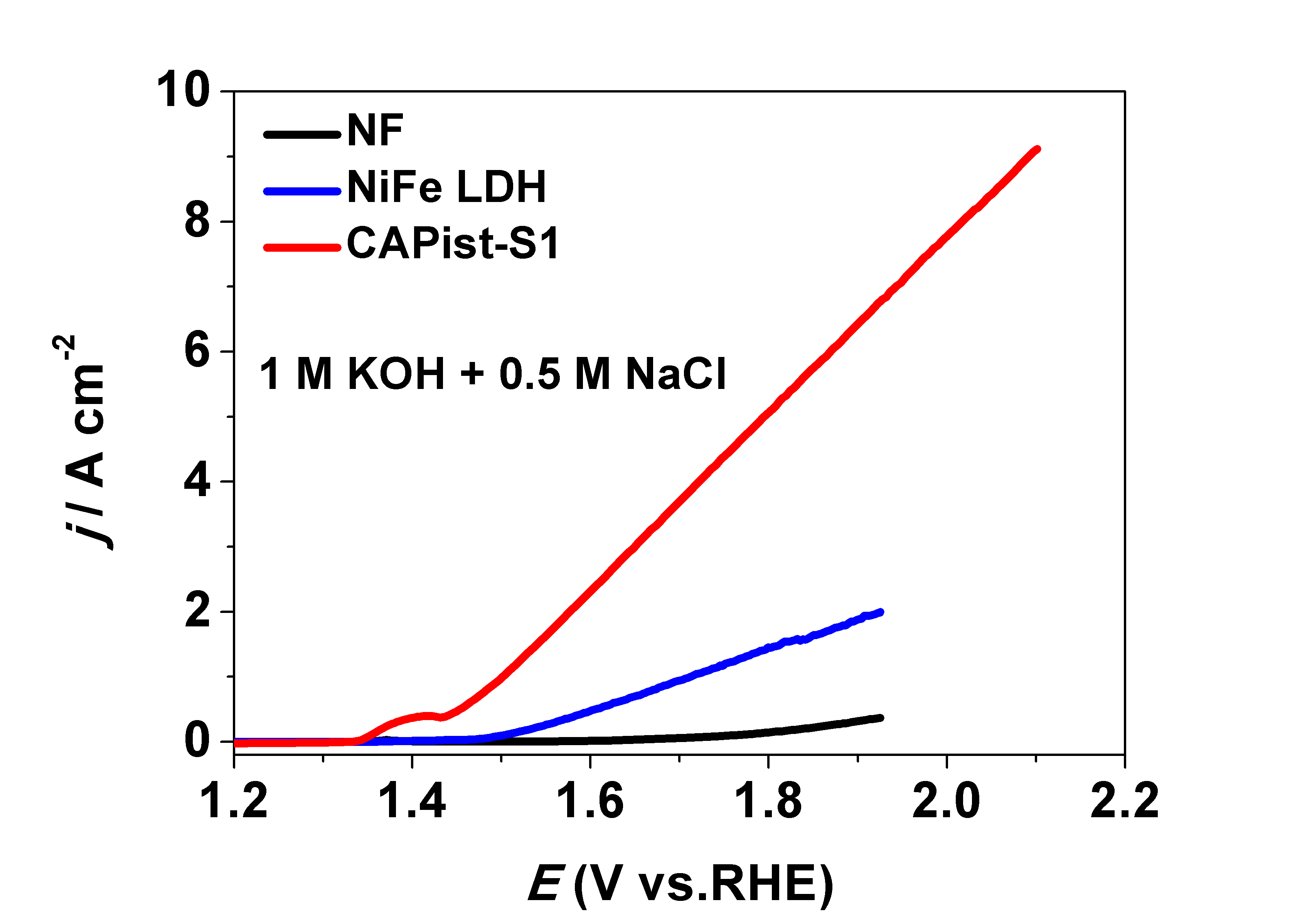


**Figure S9**. LSV curves of NF, NiFe LDH and CAPist-S1 without iR correction in alkaline simulated seawater at a scan rate of 5 mV s^-1^.


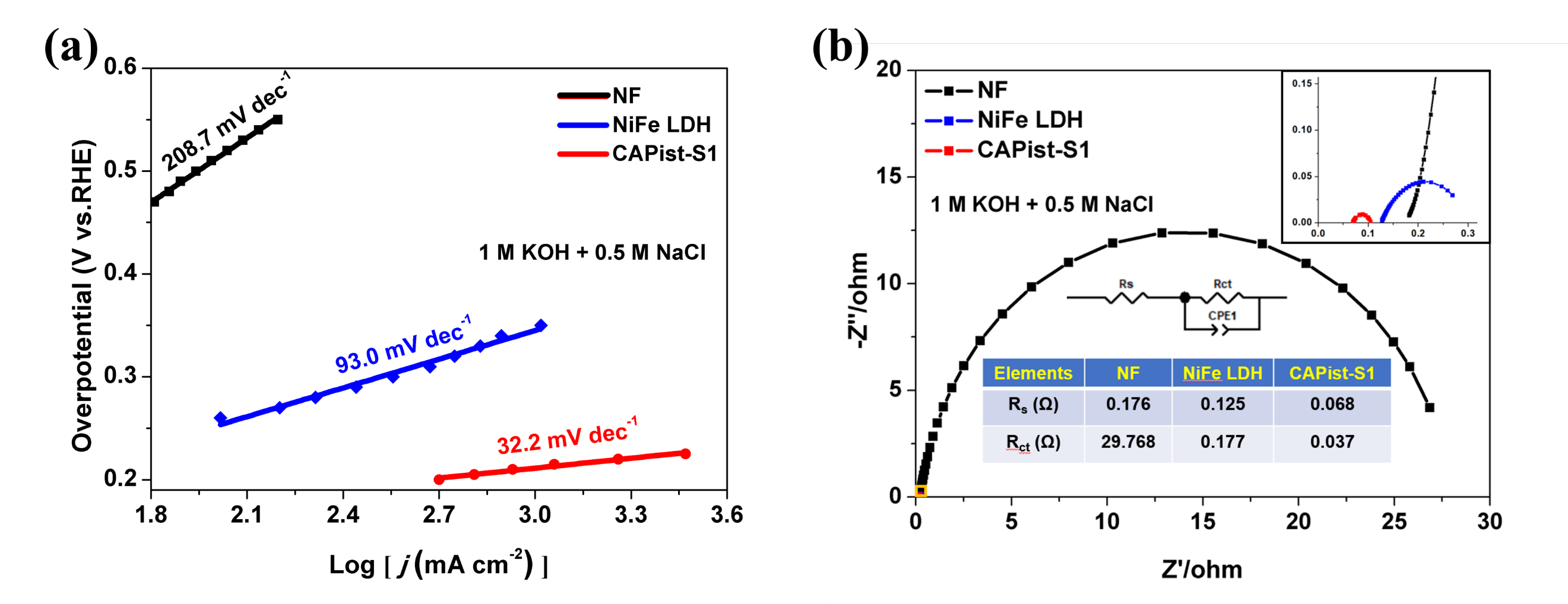


**Figure S10**. (a) Tafel plots of NF, NiFe LDH and CAPist-S1 in alkaline simulated seawater. (b) EIS Nyquist plots of NF, NiFe LDH and CAPist-S1 measured under the applied potential of 1.52 V in alkaline simulated seawater. Inset shows the enlarged region of the orange frame region, the equivalent circuit and the corresponding fitting data. The R_s_ values of NF, NiFe LDH and CAPist-S1 are determined to be 0.068, 0.125 and 0.176 Ω, respectively.


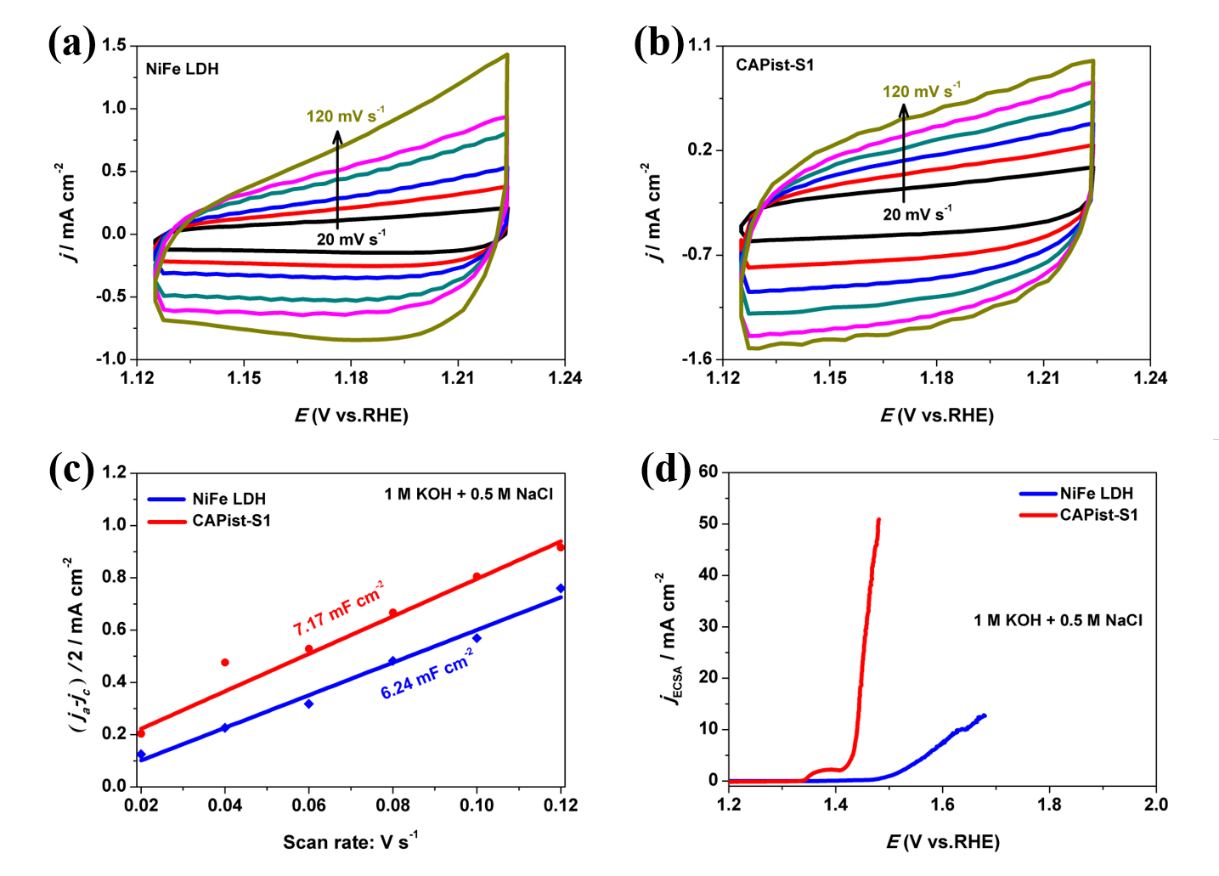


**Figure S11.** CV curves of (a) NiFe LDH and (b) CAPist-S1 at various scan rates (20 to 120 mV s^-1^ with an interval of 20 mV s^-1^) within the potential range of 0.2~0.3 V vs. Hg/HgO in alkaline simulated seawater. (c) Charging current density difference at 0.25 V vs. Hg/HgO plotted against scan rate for NiFe LDH and CAPist-S1. (d) ECSA-normalized LSV curves for NiFe LDH and CAPist-S1.


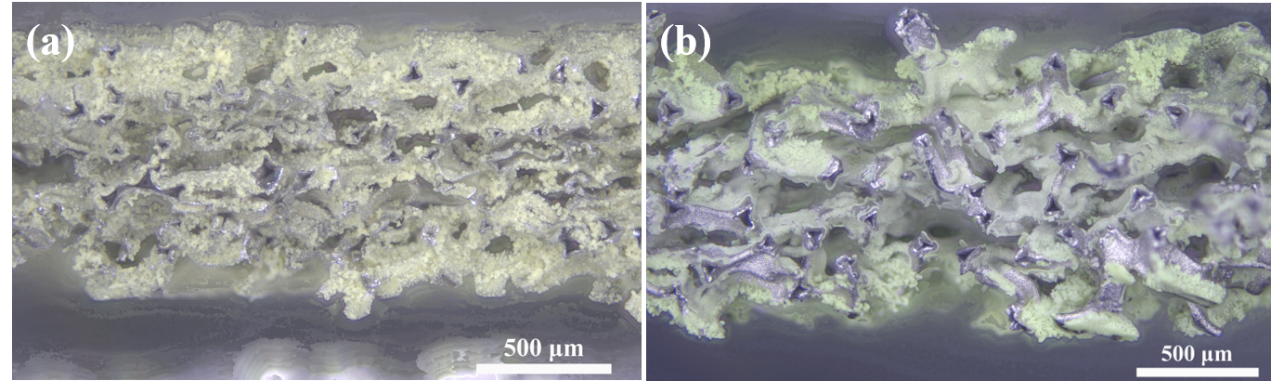


**Figure S12**. Optical images of the inner skeleton of (a) CAPist-S1 electrode and (b) NiFe LDH electrode.


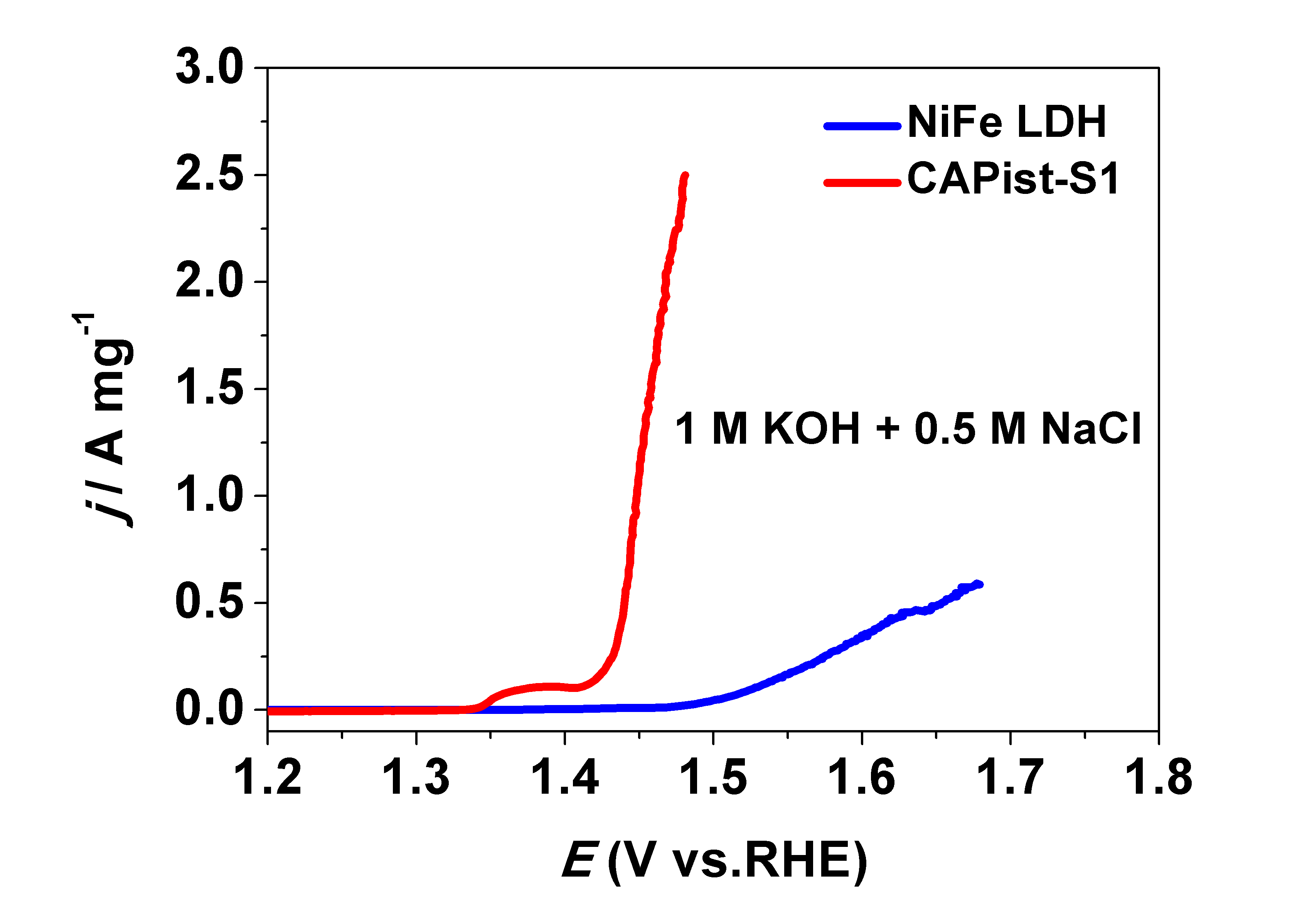


**Figure S13**. LSV curves of NiFe LDH and CAPist-S1 normalized to the loading mass of catalyst in alkaline simulated seawater.


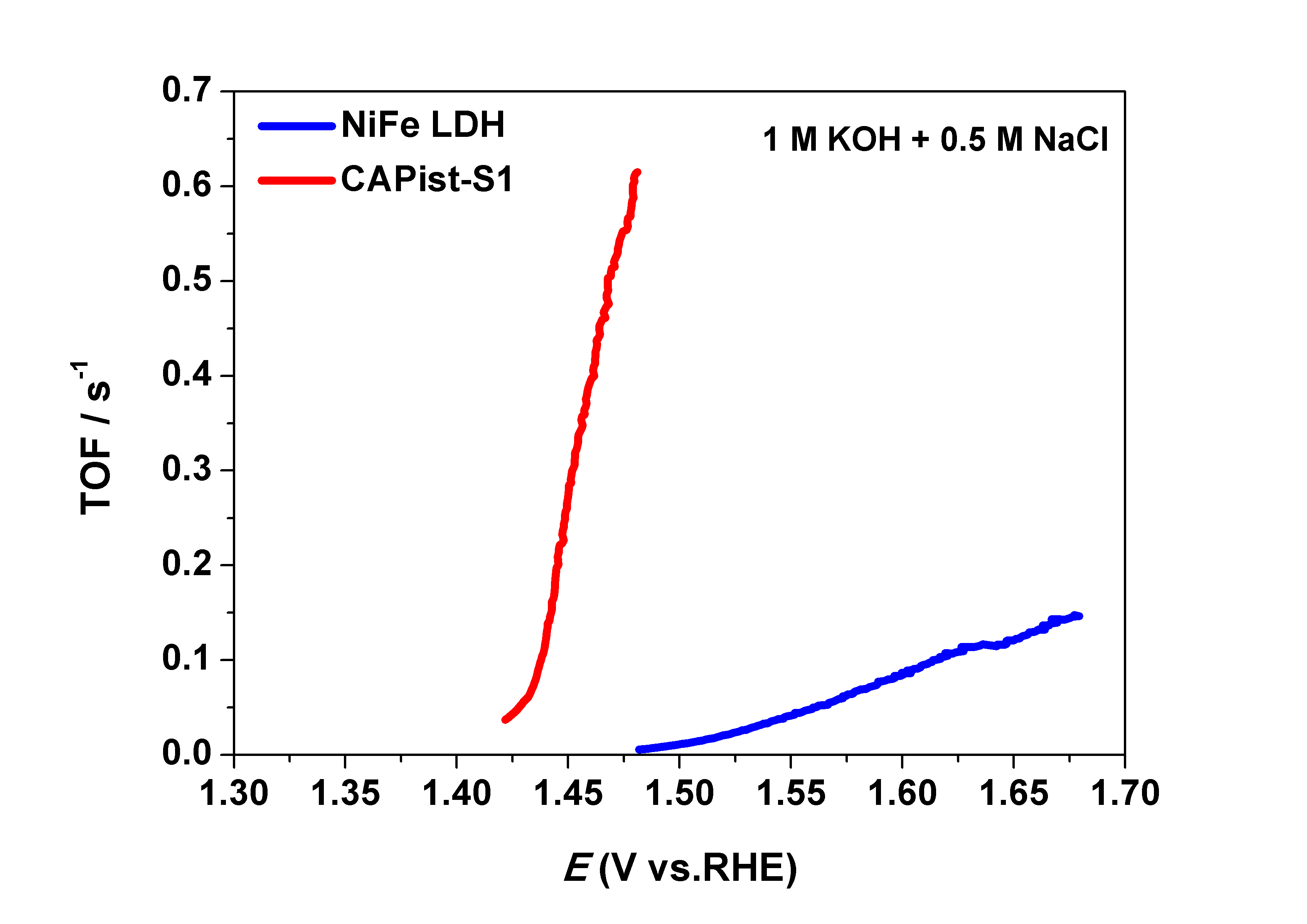


**Figure S14.** Comparison of TOF values for NiFe LDH and CAPist-S1 in alkaline simulated seawater.

*
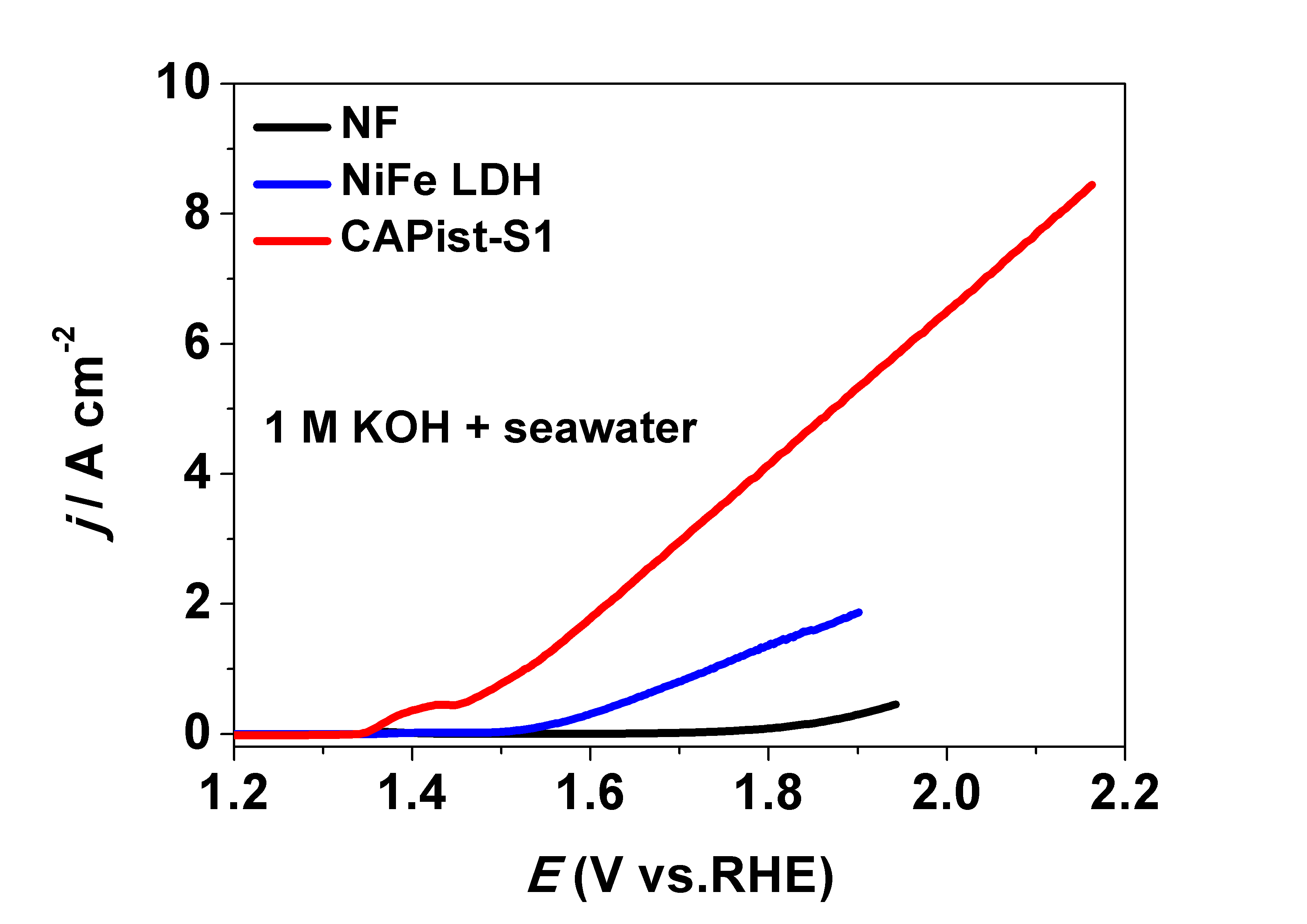
*

**Figure S15**. LSV curves of NF, NiFe LDH and CAPist-S1 without iR correction in alkaline natural seawater at a scan rate of 5 mV s^-1^.


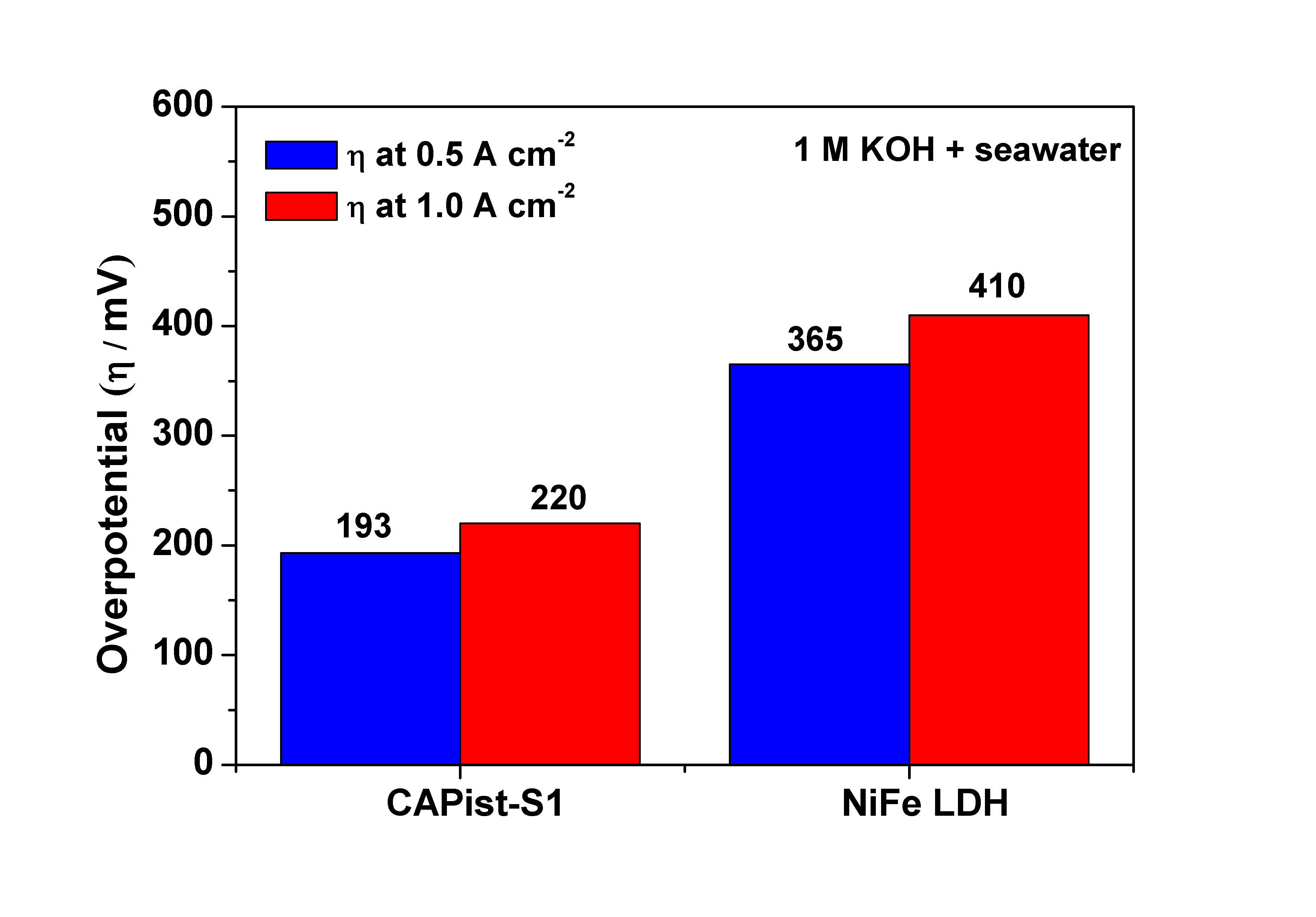


**Figure S16**. Comparison of overpotentials of CAPist-S1 and NiFe LDH at the current densities of 0.5 and 1.0 A cm^-2^ in alkaline natural seawater, respectively.


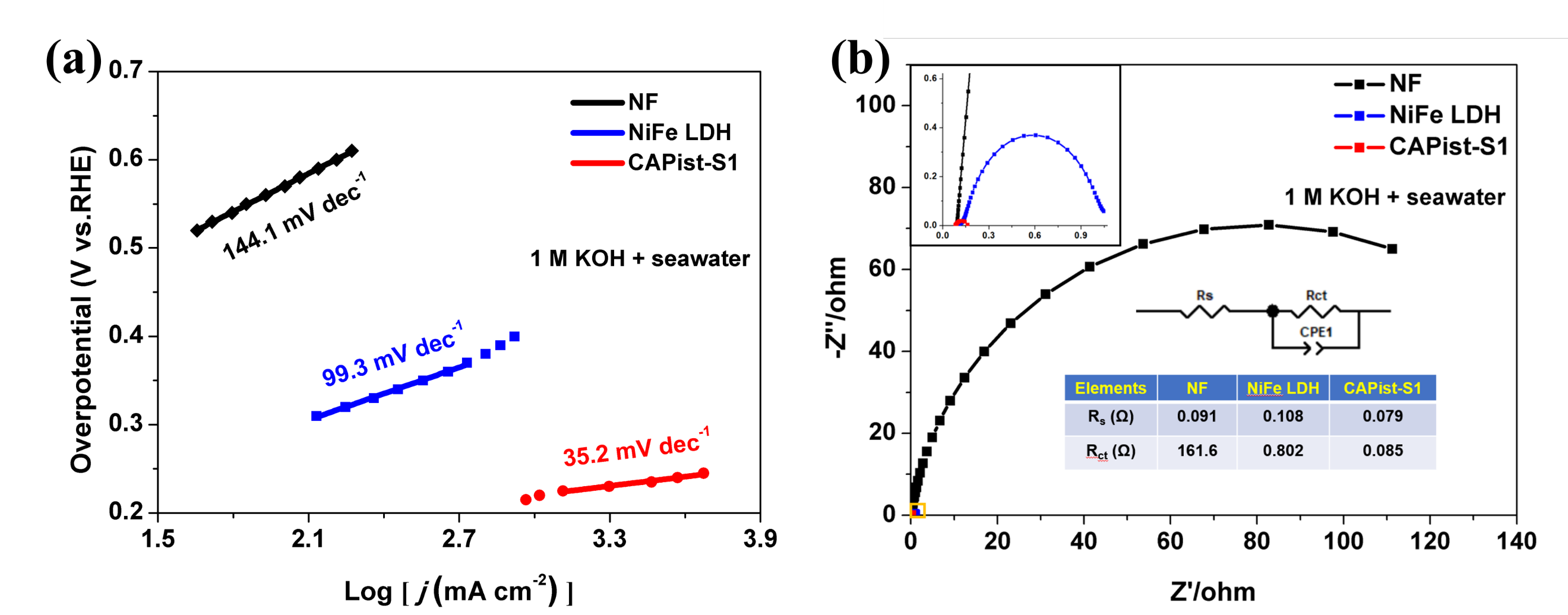


**Figure S17**. (a) Tafel plots of NF, NiFe LDH and CAPist-S1 in alkaline natural seawater. (b) EIS Nyquist plots of NF, NiFe LDH and CAPist-S1 measured under the applied potential of 1.52 V in alkaline natural seawater. Inset shows the enlarged region of the orange frame region, the equivalent circuit and the corresponding fitting data. The R_s_ values of NF, NiFe LDH and CAPist-S1 are determined to be 0.079, 0.108 and 0.091 Ω, respectively.


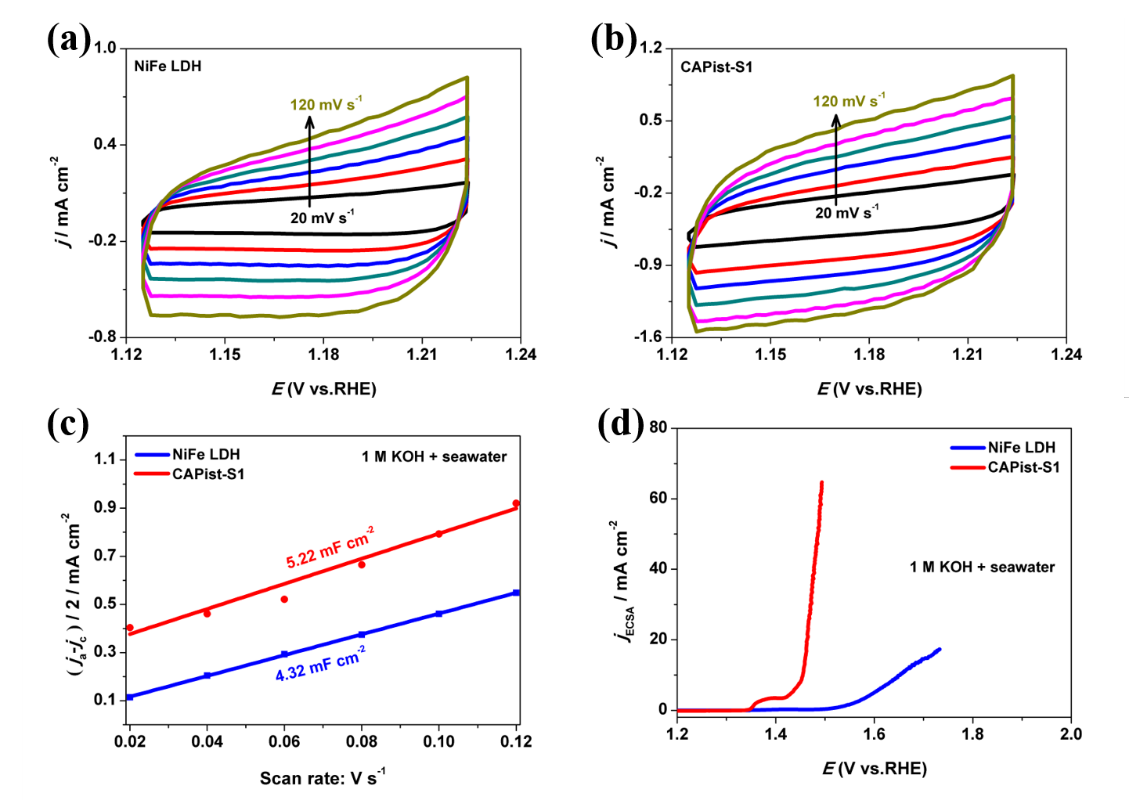


**Figure S18.** CV curves of (a) NiFe LDH and (b) CAPist-S1 at various scan rates (20 to 120 mV s^-1^ with an interval of 20 mV s^-1^) within the potential range of 0.2~0.3 V vs. Hg/HgO in alkaline natural seawater. (c) Charging current density difference at 0.25 V vs. Hg/HgO plotted against scan rate for NiFe LDH and CAPist-S1. (d) ECSA-normalized LSV curves for NiFe LDH and CAPist-S1.


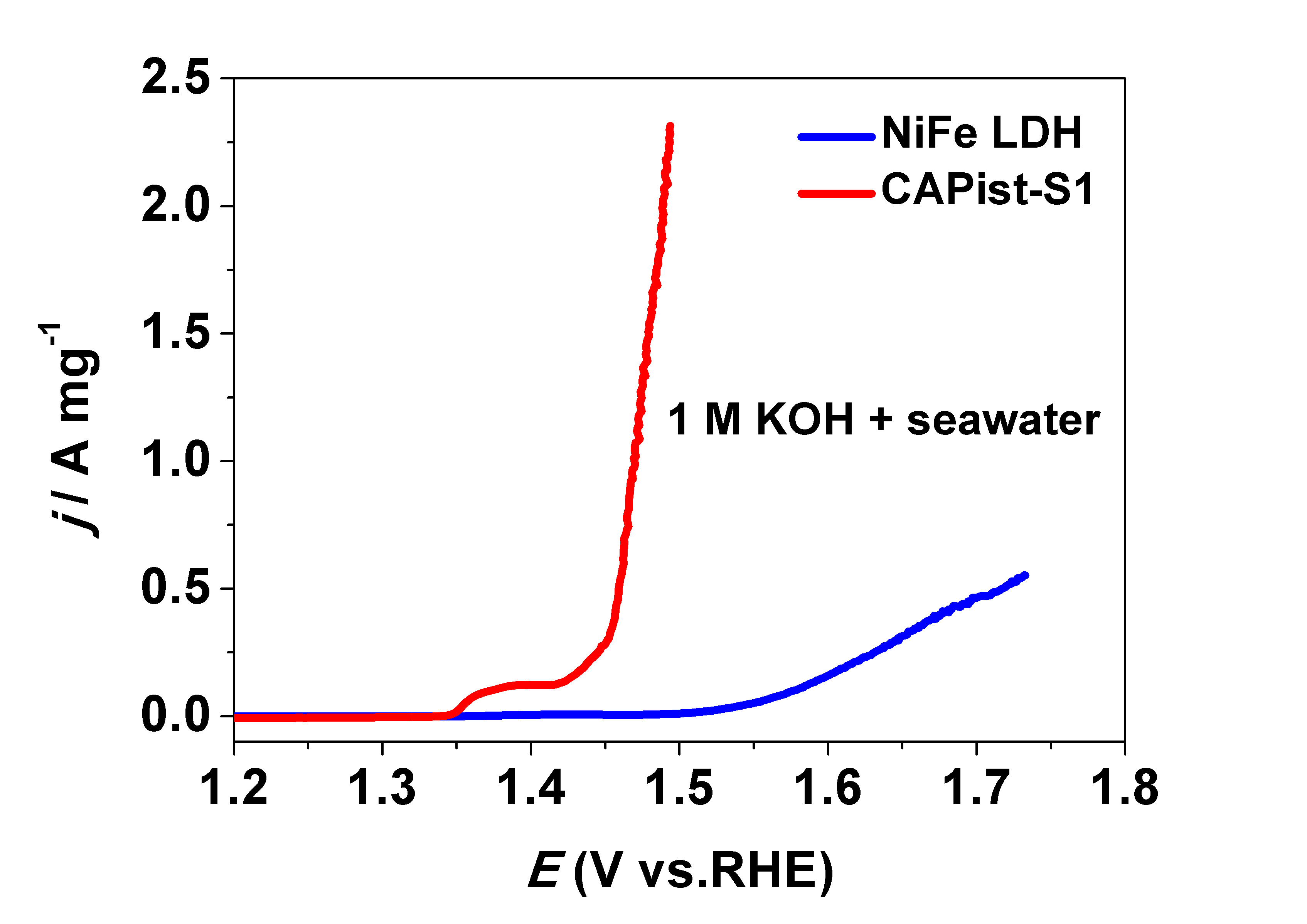


**Figure S19**. LSV curves of NiFe LDH and CAPist-S1 normalized to the loading mass of catalyst in alkaline natural seawater.


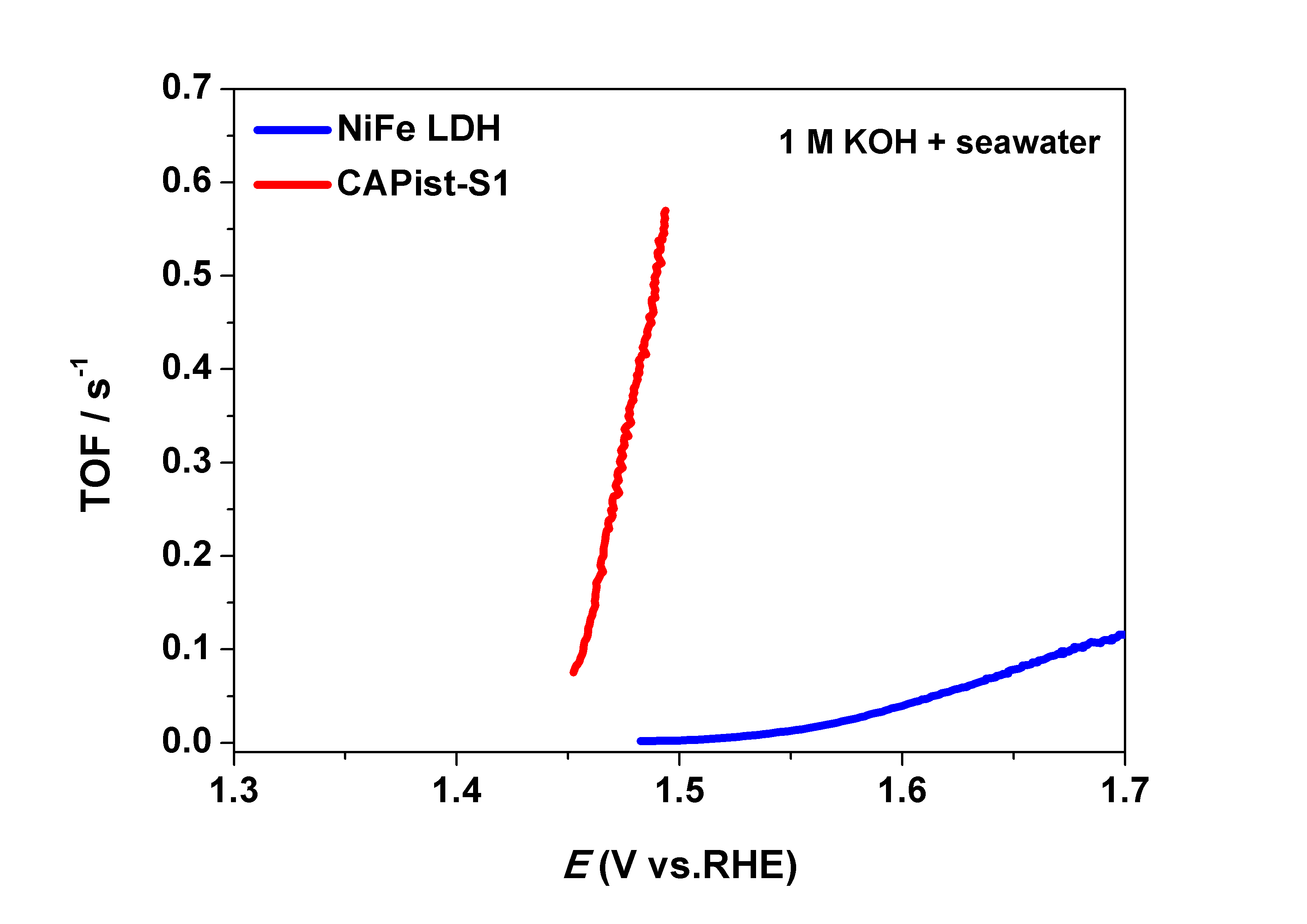


**Figure S20.** Comparison of TOF values for NiFe LDH and CAPist-S1 in alkaline natural seawater.


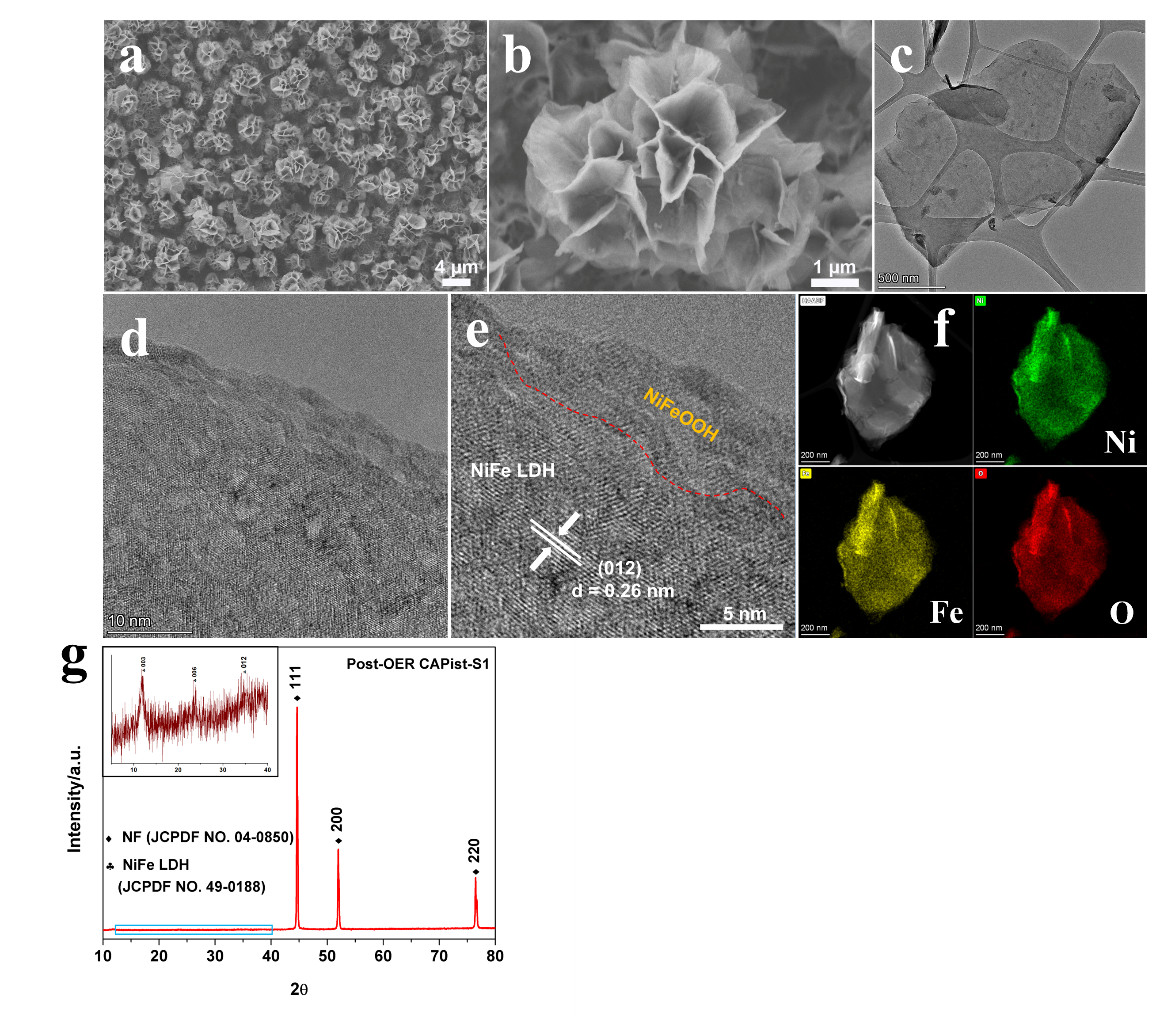


**Figure S21**. (a-b) SEM images of post-OER CAPist-S1 under different magnifications. (c-e) TEM and HRTEM images of post-OER CAPist-S1. (f) STEM image and corresponding elemental mappings of post-OER nanosheets. (g) XRD pattern of post-OER CAPist-S1. The inset shows the enlarged region in the blue frame region. As presented in Figure S21a-b, the surface morphology of NiFe LDH nanosheet maintains well after OER tests. The TEM and HRTEM images in Figure S21c-e show clear lattice fringes with an interlayer space of 0.26 nm, consistent with the (012) plane of NiFe LDH, indicating the preservation of NiFe LDH phase during OER catalysis. In addition, a dense layer with poor crystalline is observed at the edge of post-OER catalyst nanosheet, which can be ascribed to the oxyhydroxide resultant from the electrochemical oxidation. High-angle annular dark field-scanning transmission electron microscope (HAADF-STEM) image and corresponding elemental mapping in Figure S21f reveal the homogenous distribution of Ni, Fe, and O within post-OER LDH nanosheets. The XRD analysis of post-OER CAPist-S1 in Figure S21g also shows the presence of (003), (006) and (012) planes of NiFe LDH, further confirming the preservation of NiFe LDH phase during OER electrolysis.


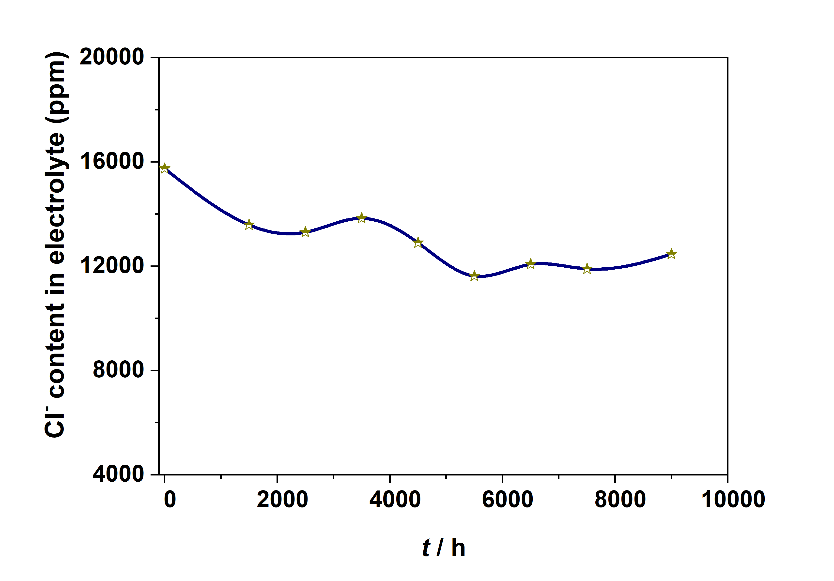


**Figure S22**. The variation of Cl content in electrolyte as a function of electrolysis time for CAPist-S1 in alkaline natural seawater.


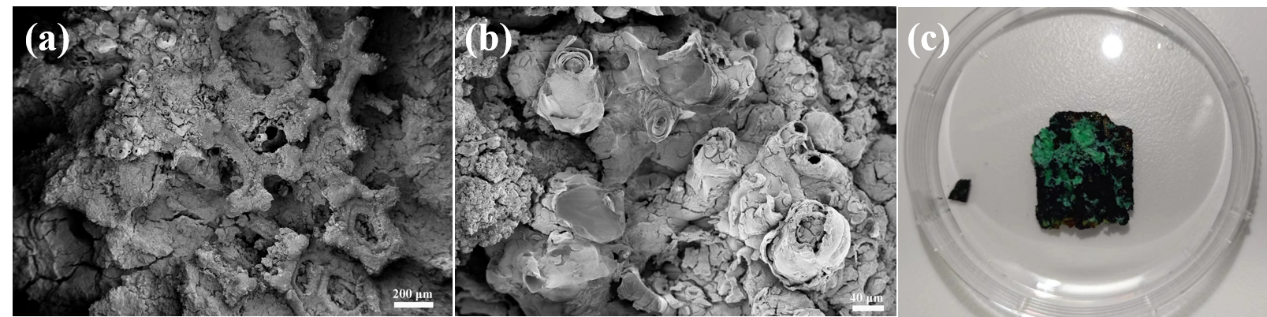


**Figure S23**. (a-b) SEM images and (c) digital photograph of NiFe LDH electrode after stability test.


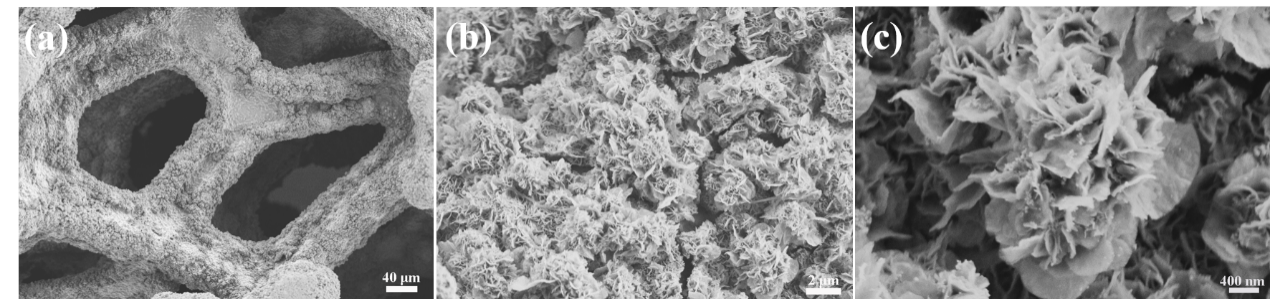


**Figure S24**. SEM images of CAPist-S1 after stability test under different magnifications.


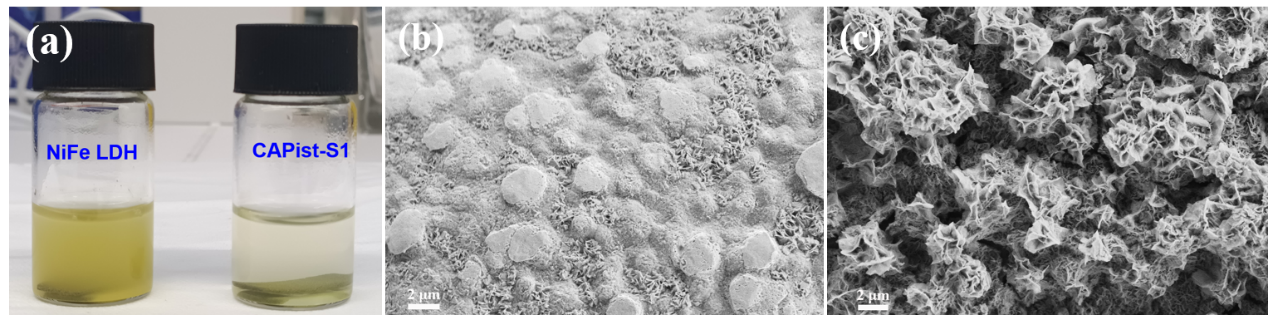


**Figure S25**. (a) Digital photograph and (b-c) SEM images of NiFe LDH and CAPist-S1 after ultrasonication treatment.


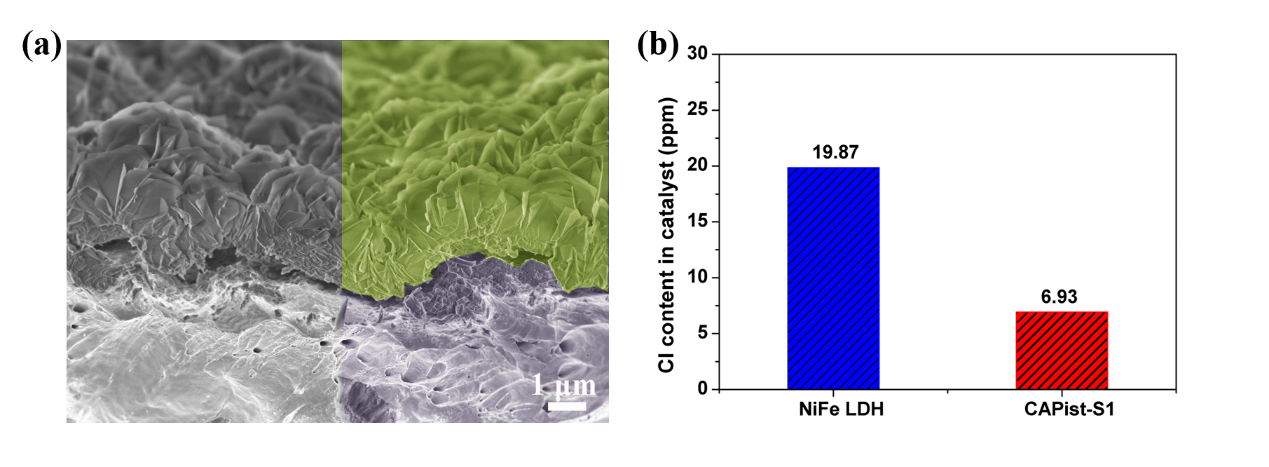


**Figure S26**. (a) Cross-sectional SEM images of NiFe LDH. (b) Comparison of Cl content in NiFe LDH and CAPist-S1 after 2 h electrolysis.


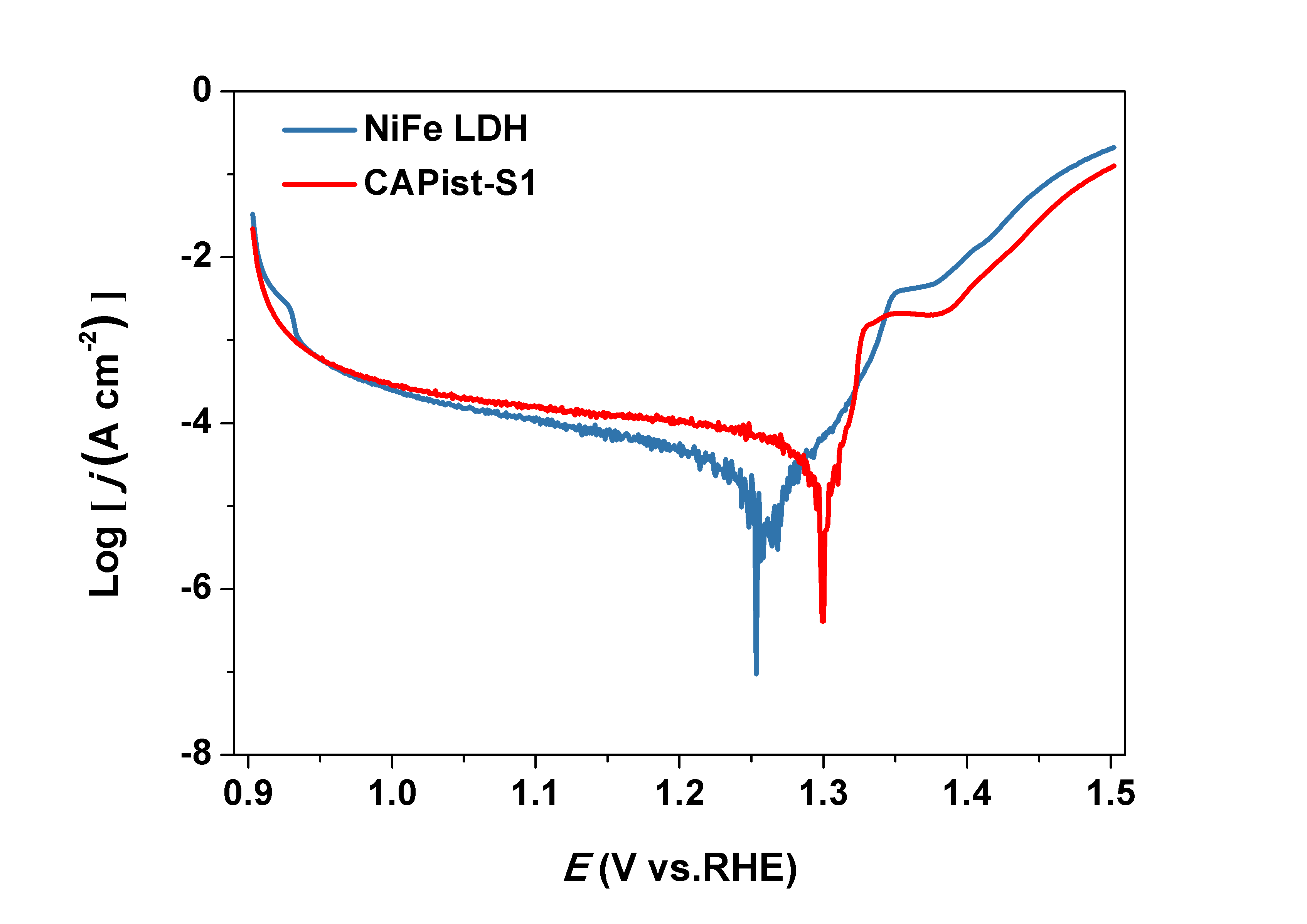


**Figure S27**. Tafel polarization curves of NiFe LDH and CAPist-S1 in alkaline simulated seawater.


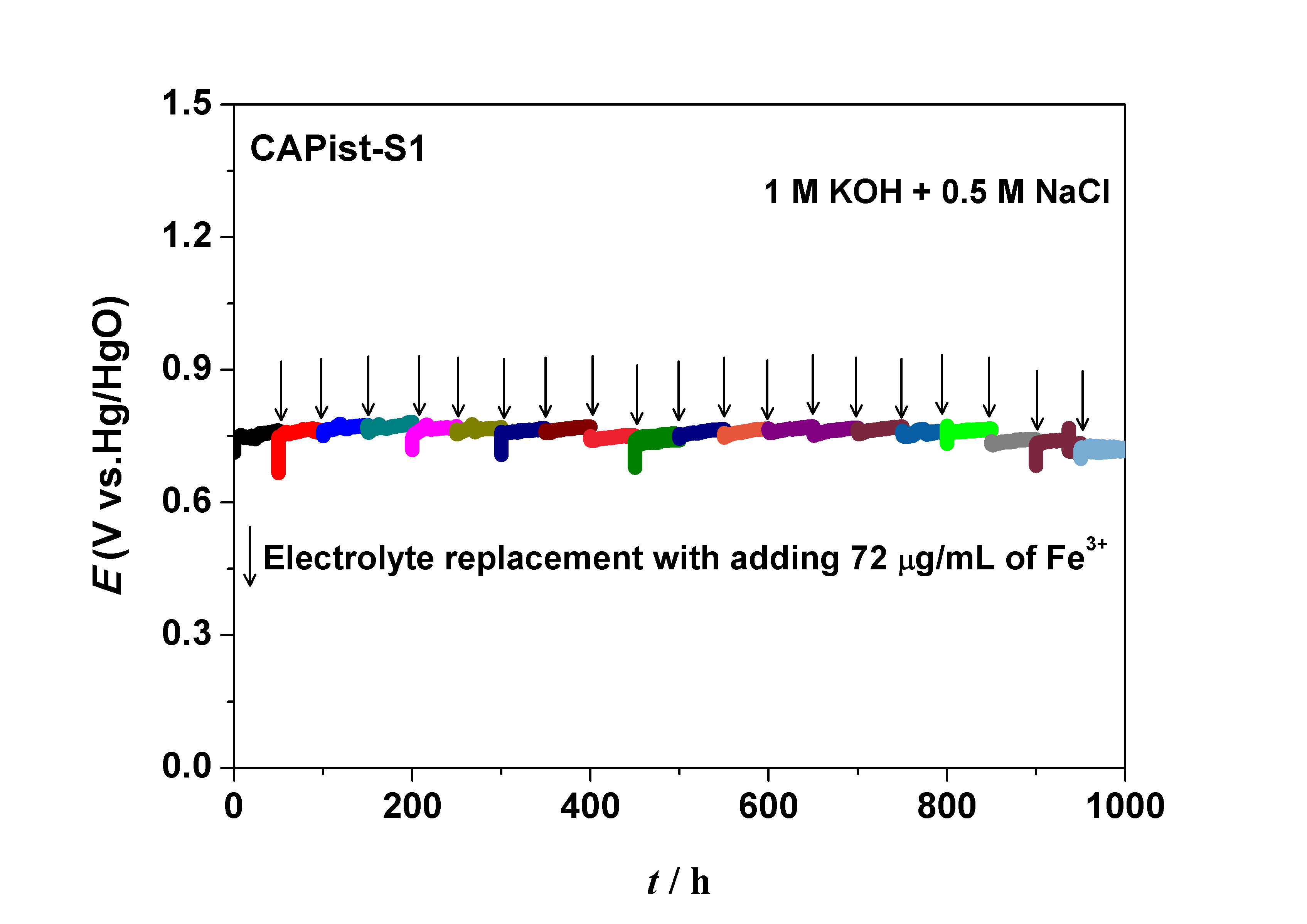


**Figure S28**. Stability tests of CAPist-S1 with Fe^3+^-added electrolyte replacement at intervals of 50 h in alkaline simulated seawater. In the experiment, small amount of Fe^3+^ (72 μg/mL) was added into the fresh electrolyte for the compensation of Fe loss from catalyst. As shown in Figure S28, the stability of CAPist-S1 was significantly prolonged when Fe^3+^ ions were added into the fresh electrolyte, revealing the CAPist-S1can sustain the stability during electrolyte replacement with the aid of externally added Fe sources.


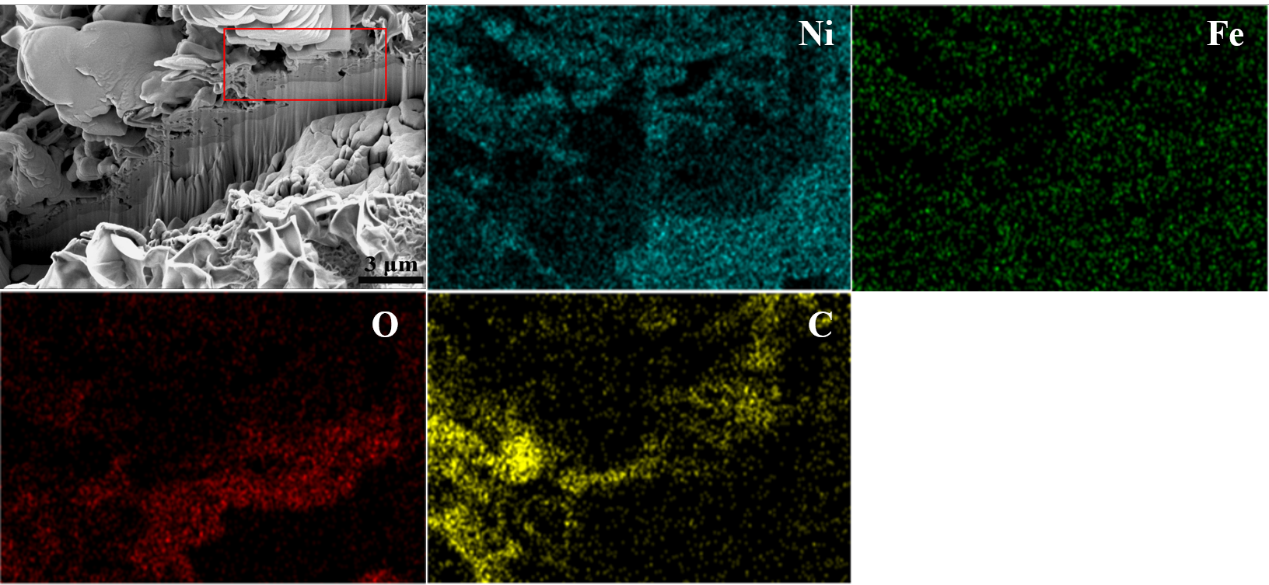


**Figure S29**. Cross-sectional SEM image of CAPist-S1 after electrolysis and the corresponding EDS elemental mapping analysis of the red frame region. After electrolysis, the CAPist-S1 electrode became relatively fragile, the stress arising from manually cutting off the electrode can lead to the severe exfoliation of catalyst layer from the substrate skeleton. Therefore, the cross section of this electrode can hardly display an integrated electrode structure. Here, to present an integrated electrode structure, focused ion beam scanning electron microscopy (FIB-SEM) was carried out. This technique works by creating an image of the surface catalyst layer, which is then stripped away using a beam of charged particles to reveal the layer beneath. The new surface can subsequently be imaged and so on, through the whole sample.


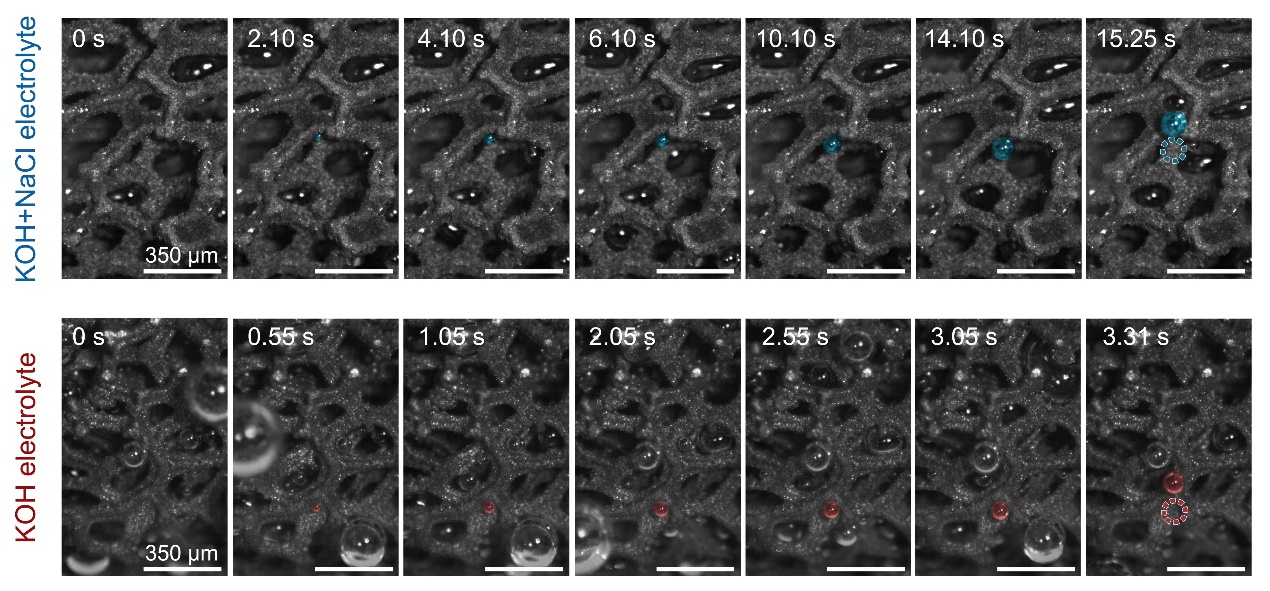


**Figure S30**. Photographs of oxygen bubble generation, growth and departure on CAPist-S1 in 1 M KOH and 1 M KOH + 0.5 M NaCl solution at the current density of 50 mA cm^-2^, respectively. A high-speed video camera was used to observe the bubble behavior on CAPist-S1 surface in diffferent electrolyte. As shown in **Figure R8**, the time from oxygen bubble generation to departure on electrode surface was determined to be 15.25 s when alkaline simulated seawater (1 M KOH + 0.5 M NaCl) was used as electrolyte, much longer than that when 1 M KOH was used as electrolyte (3.31 s), indicating the Cl^-^ ions can delay the oxygen bubble departure and thus disturb the collection of Raman signal.


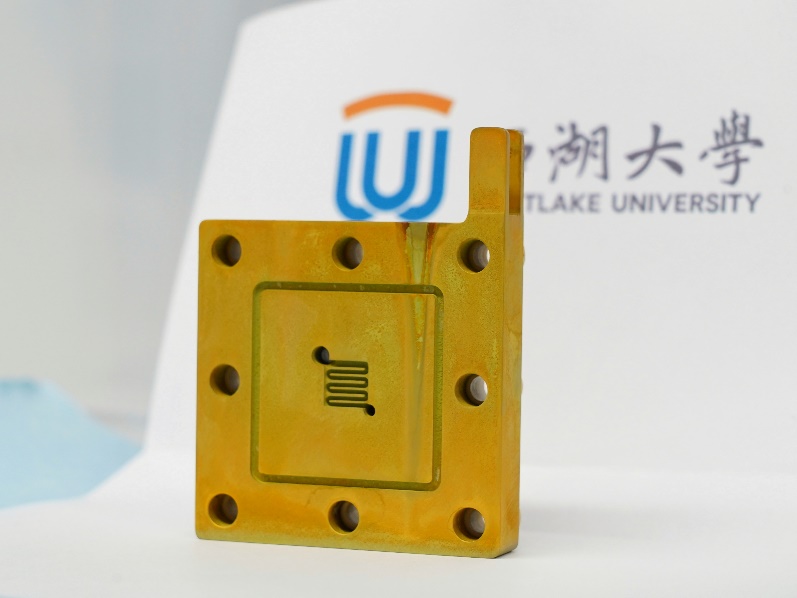


**Figure S31**. Digital photograph of Ni end plate with CAPist-S1 as protective layer.


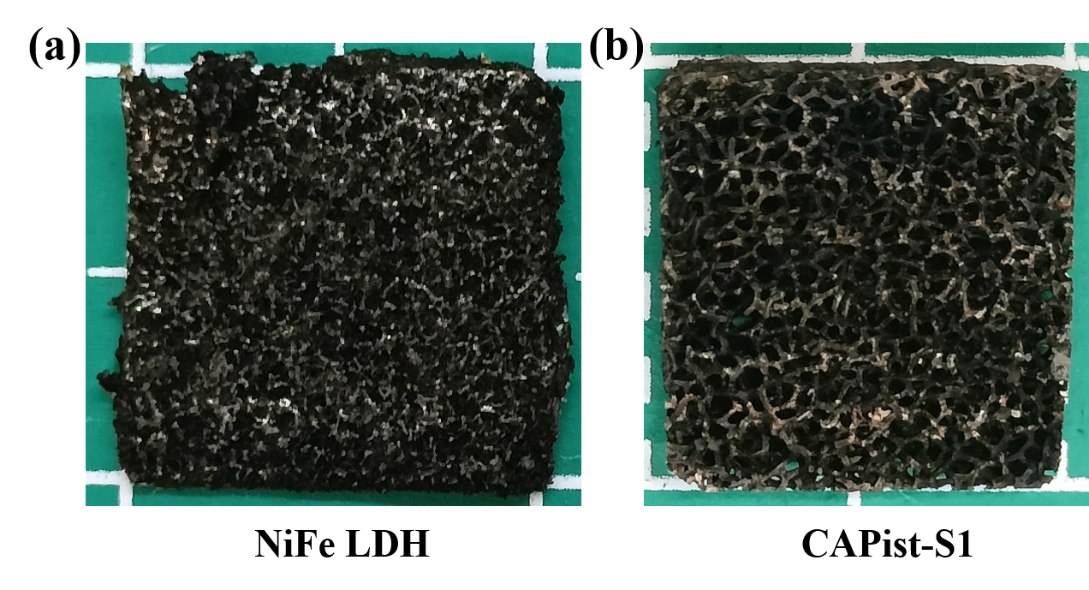


**Figure S32**. The digital photographs of NiFe LDH and CAPist-S1 after AEM seawater electrolysis.

**Table S1.** Metal content in the as-prepared CAPist-S1 derived from ICP tests.

|  | Ni/ppm | Fe/ppm |
| --- | --- | --- |
| CAPist-S1 | 69.48 | 18.00 |

**Table S2**. Comparison of the OER activities for NiFe LDH and the recently reported NiFe LDH catalysts in 1 M KOH.

| Electrocatalysts | Synthetic method | ƞ_100 mA cm-2_ | References |
| --- | --- | --- | --- |
| **NiFe LDH** | Solvothermal | **250** | **This work** |
| NiFe LDH | Hydrothermal | 283 | *Angew. Chem. Int. Ed.* **2023**, 62, e202311674 |
| NiFe-LDH | Hydrothermal | 378 | *Appl. Catal. B Environ*. **2023**, 324, 122240 |
| NiFe-LDH | Solvothermal | 332 | *Appl. Catal. B Environ*. **2021**, 284, 119740 |
| NiFe LDH | Hydrothermal | 302 | *Chem. Eng. J*. **2021**, 403, 126297 |
| NiFe-LDH | Hydrothermal | 280 | *Adv. Energy Mater*. **2021**, 11, 2101281 |
| NiFe LDHs | Hydrothermal | 280 | *Adv. Energy Mater*. **2018**, 8, 1703341 |
| NiFe-LDH | Co-precipitation | 394 | *Angew. Chem. Int. Ed.* **2021**, 60, 26829 |
| Ni_0.66_Fe_0.33_ LDH | Co-precipitation | 400 | *Mater. Chem. Phys*. **2020**, 254, 123496 |
| NiFe-WO_4_-LDH | Co-precipitation | 350 | *Sustainable Energy Fuels,* **2019**, 3, 237 |
| NiFe LDH | Electrodeposition | 290 | *Nat. Commun*. **2021**, 12, 4587 |
| NiFe LDH | Electrodeposition | 264 | *Energy Environ. Sci*., **2018**, 11, 2858 |

**Table S3**. The performance of CAPist-S1 and the recently reported OER electrocatalyst toward alkaline simulated or natural seawater oxidation.

| Electrocatalysts | Activity | | Stability | | References |
| --- | --- | --- | --- | --- | --- |
|  | Simulated | Natural | Simulated | Natural |  |
| **CAPist-S1** | **200 mV at 1.0 A cm^-2^** | **220 mV at 1.0 A cm^-2^** | / | **9000 h at 1.0 A cm^-2^** | **This work** |
| CoFe-Ci@GODs/NF | 255 mV at 0.1 A cm^-2^ | **/** | 2800 h at 1.25 A cm^-2^ |  | *Nat. Sustain*. **2024**, 7,158 |
| MoO_3_@CoO/CC | 650 mV at 0.8 A cm^-2^ | **/** | 100 h at 0.1 A cm^-2^ | **/** | *Nat. Commun*. **2024**, 15, 2481 |
| NiFeMo/NM | 296 mV at 0.1 A cm^-2^ | 322 mV at 0.1 A cm^-2^ | 1500 h at 0.1 A cm^-2^ | 550 h at 0.1 A cm^-2^ | *Adv. Energy Mater*. **2024**, 14, 2303261 |
| NiFe-LDH@Ag | 290 mV at 1.0 A cm^-2^ | / | 5500 h at 0.4 A cm^-2^ | 2500 h at 0.4 A cm^-2^ | *Adv. Mater.* **2024**, 36, 2306062 |
| NiFe-LDH_CO_3_^2-^ | 352 mV at 1.0 A cm^-2^ | / | 500 h at 1.0 A cm^-2^ | / | *Adv. Energy Mater*. **2024**, 14, 2400053 |
| RuMoNi | 470 mV at 1.0 A cm^-2^ | 484 mV at 1.0 A cm^-2^ | / | 3000 h at 0.5 A cm^-2^ | *Nat. Commun*. **2023**, 14, 3607 |
| NiFeO-CeO_2_/NF | 322 mV at 1.0 A cm^-2^ | 408 mV at 1.0 A cm^-2^ | / | / | *ACS Nano*. **2023**, 17, 16008 |
| Cr-Co_x_P | 404 mV at 1.0 A cm^-2^ | 423 mV at 1.0 A cm^-2^ | 140 h at 0.1 A cm^-2^ | 140 h at 0.1 A cm^-2^ | *Adv. Funct. Mater*. **2023**, 33, 2214081 |
| 60Fe/NF | / | 680 mV at 1.0 A cm^-2^ | / | 100 h at 0.25 A cm^-2^ | *Adv. Energy Mater*. **2023**, 13, 2301921 |
| Ni_3_N\|NiFeP/FF | 290 mV at 1.0 A cm^-2^ | / | 120 h at 0.8 A cm^-2^ | / | *Small Methods* **2023**, 7, 2201616 |
| NiS@LDH/NF | 317 mV at 1.0 A cm^-2^ | 341 mV at 1.0 A cm^-2^ | / | 100 h at 0.2 A cm^-2^ | *Small* **2023**, 19, 2300194 |
| C@CoP-FeP/FF | 317 mV at 0.1 A cm^-2^ | / | 28 h at 0.1 A cm^-2^ | / | *Small* **2023**, 19, 2206533 |
| NiOOH@FeOOH | 292 mV at 0.5 A cm^-2^ | / | 100 h at 0.5 A cm^-2^ | 100 h at 0.5 A cm^-2^ | *Adv. Mater.* **2022**, 34, 2108619 |
| Fe-NiSOH | / | 311 mV at 0.5 A cm^-2^ | / | 1100 h at 0.5 A cm^-2^ | *Energy Environ. Sci*., **2022**, 15, 4647 |
| Co_2_(OH)_3_Cl | 379 mV at 0.1 A cm^-2^ | / | ~17 h at 0.13 A cm^-2^ | / | *Adv. Funct. Mater*. **2022**, 32, 2201127 |
| CoP_x_/FeOOH | 300 mV at 0.5 A cm^-2^ | 337 mV at 0.5 A cm^-2^ | / | 80 h at 0.5 A cm^-2^ | *Appl. Catal. B Environ*. **2021**, 294, 120256 |
| S-(Ni,Fe)OOH | 378 mV at 1.0 A cm^-2^ | 462 mV at 1.0 A cm^-2^ | 100 h at 0.1 A cm^-2^ | 100 h at 0.1 A cm^-2^ | *Energy Environ. Sci*., **2020**, 13, 3439 |
| GO@Fe@Ni-Co@NF | 336 mV at 1.0 A cm^-2^ | / | 12 h at 1.0 A cm^-2^ | / | *J. Mater. Chem. A*. **2020**, 8, 24501 |
| NiFe/NiSx-Ni | 370 mV at 1.0 A cm^-2^ | / | 500 h at 1.0 A cm^-2^ | 1000 h at 0.4 A cm^-2^ | *PNAS*. **2019**, 116, 6624 |

The “simulated” and “natural” denote the electrochemical tests were performed in 1 M KOH + 0.5 M NaCl and 1 M KOH + seawater.

**Table S4**. The content of dissolved Fe in electrolyte at various time intervals during electrolysis derived from ICP tests.

| t/h | 2.1 | 7.8 | 18.4 | 31.1 | 42.1 | 52.8 | 79.1 | 95 | 104 |
| --- | --- | --- | --- | --- | --- | --- | --- | --- | --- |
| Fe/ppb | 29.83 | 135.67 | 59.1 | 16.29 | 9.19 | 10.48 | 8.16 | 5.92 | 6.16 |

**Table S5**. The performance of CAPist-S1-based AEM electrolyzer and the recently reported AEM electrolyzers toward seawater electrolysis.

| OER Cat. | HER Cat. | Membrane | Stability | References |
| --- | --- | --- | --- | --- |
| **CAPist-S1** | NF-MoO_2_/Ni_4_Mo | PiperION-A | **700 h at 1.0 A cm^-2^** | **This work** |
| Ru-Ni_2_P/Ni_5_P_4_ | Ru-Ni_2_P/Ni_5_P_4_ | / | 120 h at 0.5 A cm^-2^ | *Appl. Catal. B Environ*. **2024**, 351, 123995 |
| RuMoNi | RuMoNi | Sustainion^®^ X37-50 Grade T | 240 h at 0.5 A cm^-2^ | *Nat. Commun*. **2023**, 14, 3607 |
| NiFe LDH on Ni@Ni_x_S_y_ | CoP/C on Ni@Ni_x_P_y_ | PTPIm* | 100 h at 0.4 A cm^-2^ | *ACS Energy Lett*. **2023**, 8, 2387 |
| NiFe LDH | Raney Ni | Sustainion^®^  X37-50 Grade T | 1000 h at 0.3 A cm^-2^ | *J. Power Sources*. **2023**, 558, 232564 |
| Ni doped FeOOH | Pt/C | Sustainion^®^ X37-50 Grade T | 15 h at 0.5 A cm^-2^ | *J. Mater. Chem. A*. **2021**, 9, 9586 |

All the electrochemical tests were performed by using 1 M KOH + seawater as circulated electrolyte. Catalyst was abbreviated as ‘Cat’ in the table.
